# Object permanence in rooks (*Corvus frugilegus*): Individual differences and behavioral considerations

**DOI:** 10.3758/s13420-024-00637-0

**Published:** 2024-09-03

**Authors:** Francesca M. Cornero, Nicola S. Clayton

**Affiliations:** Department of Psychology, University of Cambridge, Downing Street, Cambridge, CB2 3EB UK

**Keywords:** Birds, Object permanence, Comparative psychology, Corvids, Rooks, Secondary representation

## Abstract

Piagetian object permanence (OP) refers to the ability to know that an object continues to exist when out of sight: In humans, it develops in six stages. Species of great apes, other mammals, and birds (parrots, corvids, and pigeons) have been shown to possess partial or full OP, which is a prerequisite for more complex physical cognition abilities they may possess. In birds, the greatest variation is in Stage 6 (invisible displacements) and in “A-not-B” errors—incorrectly persevering in searching an empty location rewarded previously. Caching abilities have been invoked as holding explanatory power over results in corvids, for which this error is sometimes completely absent. The rook (*Corvus frugilegus*), a cognitively advanced, social, caching corvid, has not yet been studied for OP. This study applies tasks of one OP scale commonly adapted for nonhuman animals, Uzgiris and Hunt’s Scale 1, as well as later-conceived tasks 16 and S, to a sample of adult, captive rooks. One rook demonstrated full OP (Stage 6b, multiple invisible displacements), whereas other individuals varied, attaining between Stages 5a (single visible displacements) and 6a (single invisible displacements). Like some corvids, a few made transient “A-not-B” errors. Behavioral considerations potentially underlying observed individual variation in results in rooks, including dominance, neophobia, past experiences, and individual idiosyncrasies, are examined. Rooks, like other corvids, possess well-developed OP abilities, and these results support the idea that exertion of executive control is required to avoid “A-not-B” errors, rather than caching abilities or developmental age, as previously suggested.

**Supplementary Information:**

The online version contains supplementary material available at 10.3758/s13420-024-00637-0.

## Introduction

**Fig. 1:**
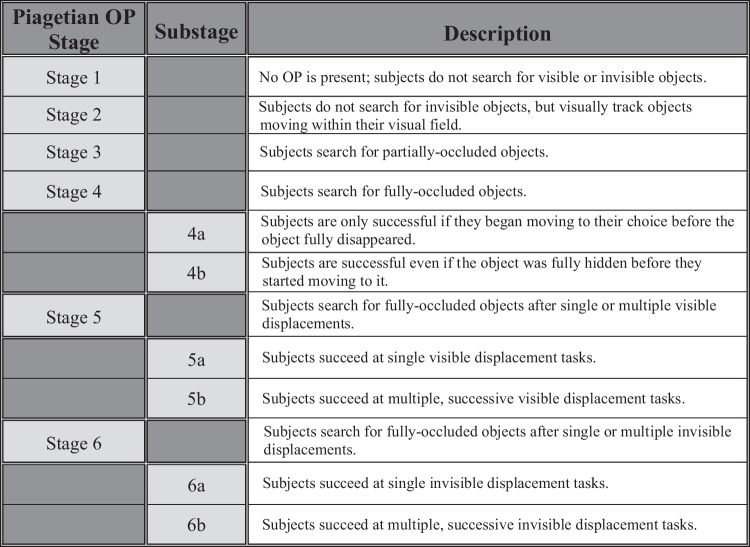
Piagetian OP stages, substages, and descriptions; compiled as reviewed by Zewald and Jacobs (2021)

Object permanence (OP) refers to the ability to conceive that an object exists in real space even when out of sight: Humans develop this ability by 2 years of age in a well-characterized, step-wise manner (Piaget, 1952, 1954). According to Piaget, it comprises six stages (Fig. 1): In Stage 1, infants do not track moving objects or notice when they are then hidden. In Stage 2, they track moving objects but do not search for objects placed out of sight. In Stage 3, older babies can retrieve objects that have been partially occluded, whereas in Stage 4 they can retrieve them when fully occluded. In Stage 5a, subjects can find a hidden object if it was visibly displaced once, but are vulnerable to “A-not-B errors”: continuing to search where an object was previously hidden repeatedly (Location A) though it is now hidden elsewhere (Location B). Subjects must stop this error before proceeding, and in Stage 5b they can find a hidden object if it was visibly displaced more than once. Finally, in Stages 6a and 6b, subjects can find objects that were invisibly displaced one or more times—that is, objects that were not directly seen again during the displacements (see Zewald & Jacobs, 2021, for a review).

**Fig. 2:**
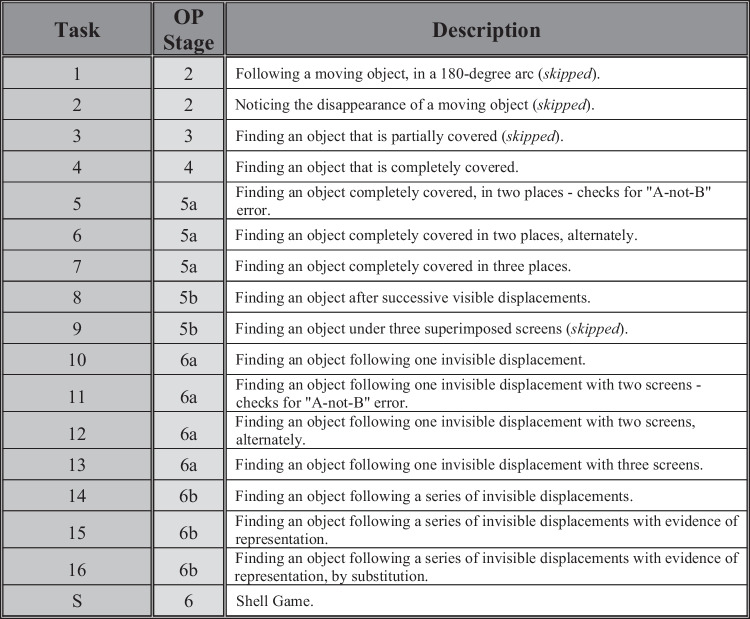
Conceptual descriptions of Uzgiris and Hunt’s (1975) Scale 1 tasks (plus Tasks 16 and S), with associated OP stages; compiled with input from Pepperberg and Kozak (1986); Pepperberg et al. (1997); Pollok et al. (2000); Zewald and Jacobs (2021); Zucca et al. (2007). Tasks noted as “skipped” were not administered in this study

The Piagetian framework is tractable for studies with nonhuman animals: It does not require verbal responses and is relatively simple to administer (Chevalier-Skolnikoff, 1976; Pepperberg et al., 1997). One iteration, Uzgiris and Hunt’s (1975) Scale 1, has repeatedly been adapted for animals (see Zewald & Jacobs, 2021): It is a series of 15 tasks, with corresponding success criteria, targeting specific OP stages incrementally (see Fig. 2 and the Supplementary Materials). It is often complemented with two additional tasks, 16 and S, which further target invisible displacements (see Doré et al., 1996; Pepperberg et al., 1997; Sophian, 1985). Using this, several animals have demonstrated some level of OP, including full Stage 6; of particular interest here are great apes and birds, especially parrots and corvids (e.g., *great apes*, Call, 2001; *carrion crows*, Hoffmann et al., 2011; *grey parrots*, Pepperberg & Funk, 1990; Pepperberg et al., 1997; *magpies*, Pollok et al., 2000; *jackdaws*, Ujfalussy et al., 2013; *Eurasian jays*, Zucca et al., 2007; *Western scrub-jays*, Salwiczek et al., 2009; see Supplementary Materials for more).

Most often, the onset of OP during development is studied, so subjects are studied from a young age and offered tasks repeatedly to establish age of competence (e.g., Pepperberg et al., 1997; Pollok et al., 2000; Salwiczek et al., 2009, among others), but occasionally adults are examined (e.g., Mathieu et al., 1976; Pepperberg & Kozak, 1986; Pepperberg et al., 1997; Triana & Pasnak, 1981). Over time, studies have incorporated controls for alternative explanations, including olfaction, unconscious cueing, local enhancement (e.g., “search last blind touched”), and baiting order (e.g., “search blind closest to occluder”; e.g., Jaakkola, 2014; Zewald & Jacobs, 2021). Not all studies control for all possibilities—this particularly affects invisible displacements (Jaakkola, 2014), which are particularly interesting due to their level of difficulty.

Tracking invisible displacements (Stage 6) is particularly challenging to demonstrate, for both subjects (for which it is likely cognitively difficult) and researchers (who must exclude several alternative explanations). At minimum, passing invisible displacements requires understanding that an object can move out of sight, being found in a location other than that where it first disappeared (see Zewald & Jacobs, 2021). At most, it may require entertaining secondary representations (Suddendorf & Whiten, 2001) and possibly even rudimentary inferential abilities (Jaakkola, 2014). This is because, to track and locate an object that was displaced invisibly, subjects may need to entertain both the representation of what was last visible (when the object was first hidden) and the representation of where it is logically found at retrieval, after invisible transfers (Suddendorf & Whiten, 2001). Primary representation would not suffice because neither the visible state nor past representation alone provide a solution to the problem, and the representation of past events must be understood as being equally about the problem at hand as the nonvisible representation of where the object must have been moved (Suddendorf & Whiten, 2001). Additionally, it may require some inferential reasoning in tasks for which logic is required to find the solution (e.g., Tasks 10–13, where the intermediate occluder is shown to be empty, and the only possible location of the item must be, to some extent, inferred; Jaakkola, 2014; Klerk & Jacobs, 2021; Zewald & Jacobs, 2021). However, it is possible subjects may simply continue to search in out-of-sight places, rather than understanding with certainty the object *must* be in the correct location with (for a discussion of certainty vs. possibility in inferential reasoning, see Pepperberg et al., 2019).

Another behavior of interest is the well-characterized occurrence of the common “A-not-B” error in Tasks 5 and 11 of Scale 1 (Uzgiris & Hunt, 1975). These tasks test for this common error by baiting one location repeatedly (at least twice, visibly in Task 5 and invisibly in 11) and then switching to another location during a following trial. Many, but not all, individuals of many species will initially display the “A-not-B” error, persisting in searching the location that had been multiply reinforced rather than the location they have witnessed the object being moved to (for a discussion, see Osthaus, 2021; Zewald & Jacobs, 2021). The mechanisms underlying “A-not-B” errors are not entirely understood; in humans, the initial occurrence of these and their cessation was thought to signify strengthening OP abilities with developmental age (Osthaus, 2021; Piaget, 1952, 1954). However, studies with adult animals that initially make these errors then cease (i.e., *African grey parrots*, Pepperberg & Kozak, 1986; Pepperberg & Funk, 1990), and some developing animals that do not make these errors (*magpies,* Pollok et al., 2000; *Eurasian jays,* Zucca et al., 2007; to some extent, *jackdaws,* Ujfalussy et al., 2013) suggest maturing OP abilities alone cannot account for all errors. Immature spatial or allocentric coding systems have been suggested as a cause instead (see discussion in Pollok et al., 2000), but, again, the observed results do not support this explanation alone, since flighted birds, including all who made the errors, require these systems to be mature and sophisticated for successful flight (Pollok et al., 2000; Wang et al., 2021). Finally, executive function may underlie the “A-not-B” error, as it may be necessary to inhibit a previously reinforced behavior when it is no longer appropriate (see Gómez, 2005; Osthaus, 2021; Wang et al., 2021; among others).

The ability to cache has also been suggested to have explanatory value for the absence of “A-not-B” errors in some corvids. Several corvids cache food for winter (Vander Wall, 1990), an adaptation likely evolved to counteract predictable periods of scarcity: While some cache for a few days, others recover food after months (de Kort et al., 2006; Roberts, 1979). Studies have shown an important contribution of spatial memory in cache recovery (Kamil & Balda; 1985; Kamil et al., 1999; Krebs et al., 1996), and caching birds appear to perform better on other spatial memory tasks, and preferentially attend to spatial cues, than noncaching birds, though they do not necessarily have better general memory (Brodbeck, 1994; Clayton, 1992, 1994; Clayton & Krebs, 1993, 1994a; Healy et al., 1994; Healy & Krebs, 1992a; Kamil et al., 1994; Krebs, 1990; Krebs et al., 1996; Olson et al., 1995). Caching behavior correlates with hippocampal enlargement (Garamszegi & Eens, 2004; Healy et al., 1994; Healy & Krebs, 1992b, 1993, 1996; Krebs, 1990; Lucas et al., 2004), hippocampal lesions affect cache recovery (Sherry & Vaccarino, 1989), and preventing individuals from caching in development caused the hippocampus to remain smaller (Clayton & Krebs, 1994b). Resistance to memory interference due to caching abilities may underlie the lack of “A-not-B” errors in some species, such as magpies and Eurasian jays (Clayton & Krebs, 1994a; Hampton & Shettleworth, 1996; see discussion in Pollok et al., 2000; Zucca et al., 2007). However, that other caching corvids, such as ravens and carrion crows, initially make “A-not-B” errors would then be surprising, and still require additional explanation, because resistance to interference due to ecologically relevant caching abilities should also apply there (see Wang et al., 2021).

Rooks (*Corvus frugilegus*) are a highly social and colonial corvid species (Emery et al., 2007; Seed et al., 2007), often mating for life and returning to nesting sites yearly (Seed et al., 2007). In the wild they cache extensively, and recover caches accurately after months (Dally, Clayton, et al., 2006a; Källander, 2007). They exhibit complex social behaviors: social foraging (Dally et al., 2008; Jolles et al., 2013), third-party postconflict affiliation (Emery et al., 2007; Seed et al., 2007), possibly recognizing their partners in video (Bird & Emery, 2008), and cooperate with conspecifics to solve string-pulling problems (but without evidence of understanding underlying causalities; Seed et al., 2008). They have solved physical problems, including tool use, tool modification, metatool use, and show some understanding of physical cognition (Bird & Emery, 2009a, 2009b, 2010; Seed et al., 2006, 2008; Tebbich et al., 2007). It would seem logical that rooks must have sophisticated OP abilities as well, but no data are available. Presenting OP tasks to rooks, a caching corvid, would also further examine the effect of caching on “A-not-B” errors and OP abilities as a whole, which are likely required for successful caching (Bugnyar et al., 2007; Pollok et al., 2000; Salwiczek et al., 2009; Zucca et al., 2007).

In this study, most of Uzgiris and Hunt’s (1975) Scale 1 was applied to an adult, captive sample of rooks, some of which had participated in experiments mentioned previously (see the Methods and Discussion sections). Additionally, Tasks 16 and S were also presented for additional investigation into invisible displacements. It was predicted that rooks should have complex OP abilities due to their caching and other physical cognition abilities.

## Methods

### Subjects

The sample was eight adult rooks (15–20 years old at the start of testing): Leo, Connelly, Plato, Aristotle (males); Fry, Bussell, Cassandra, Huxley (females). Cassandra, Aristotle, and Bussell never became willing to work, resulting in *n* = 5. Rooks lived as a group in a large outdoor aviary (20 × 10 × 3 m): Smaller indoor compartments (3 × 1 × 2 m), connected by hatches (0.5 × 0.5 m), were used for testing (see Supplemental Fig. 1A). Testing began on March 30, 2021; Leo was tested until August 2021; Fry and Connelly were tested until February 2023.

Rooks were taken from wild colonies around the laboratory as nestlings between the years 2002 and 2006 with appropriate Natural England licenses, were hand-raised by Clayton and her lab, and had subsequently lived in laboratory settings: Several had participated in some of the experiments mentioned above (see Supplementary Materials). The experiment was approved by the University of Cambridge (AWERB Sub-Standing Committee) as a nonregulated procedure (NR2023/45), and followed Home Office Regulations and the Association for the Study of Animal Behaviour’s Guidelines for the Treatment of Animals in Behavioural Research and Teaching.

A long habituation period (to experimenter F.M.C., testing compartments, and *single cups only*) was necessary before birds became willing to approach and work (about 100 habituation days; see the Discussion section). Subsequently, during testing sessions, which were as long as a subject was willing to participate and always began and ended by the bird voluntarily approaching or leaving the testing area, individuals were visually isolated from other rooks when possible. Although the rooks being tested could not be shut inside the testing compartments for visual isolation from other rooks (their neophobia towards this procedure could not be overcome), rooks in the aviary were not able to observe trials unless they approached the area immediately outside the compartment, and if so, the experiment was paused until they left. Birds were never food or water deprived. Birds were fed a maintenance diet of soaked cat biscuits, vegetables, seeds, fruit and hard-boiled eggs. Waxworm larvae, a highly preferred food not otherwise available, were used as experimental treats and as the objects to be retrieved.

### Apparatus

**Fig. 3:**
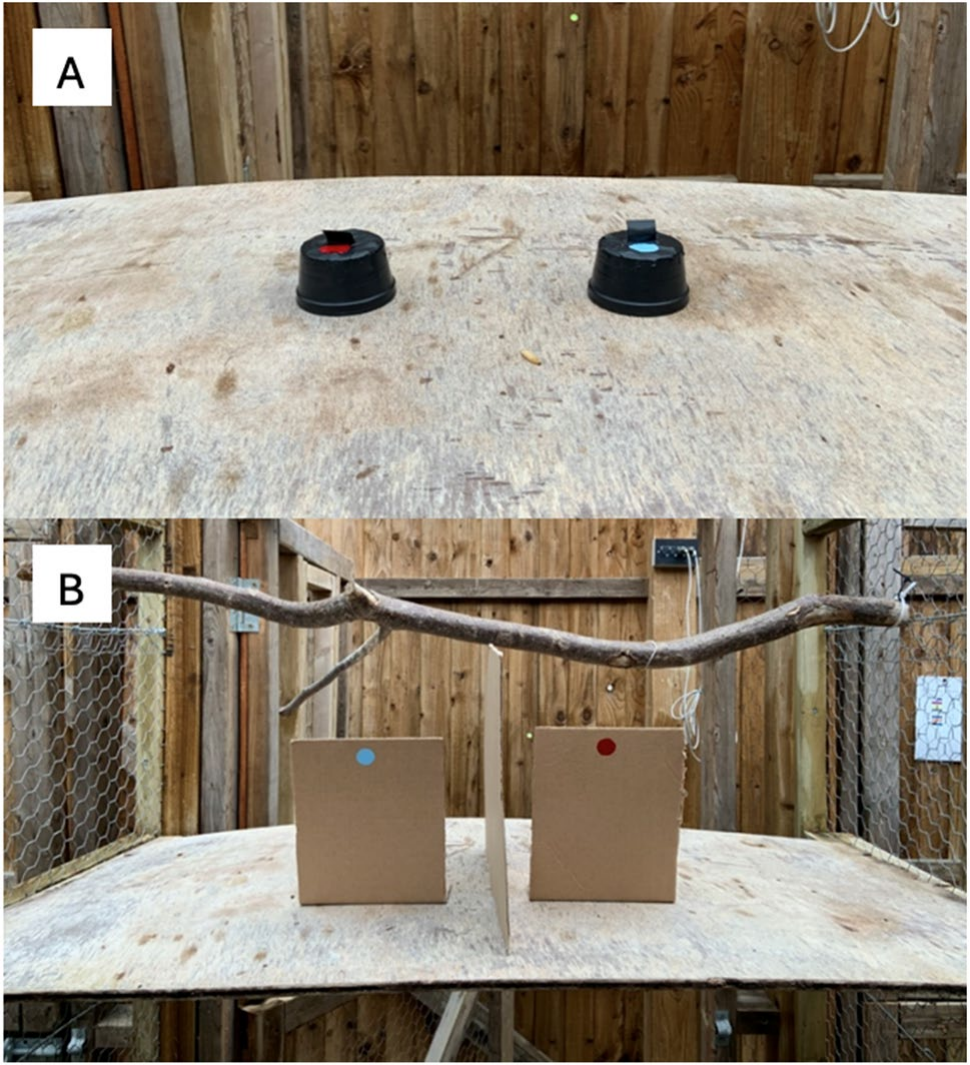
**A** The cups used as blinds for Tasks 4–8 and S (pictured, red and blue cups, from the bird’s perspective, as well as a waxworm). **B** The cardboard barriers used as blinds for Tasks 10–16 (pictured, red and blue blinds, from the bird’s perspective). (Color figure online)

**Fig. 4:**
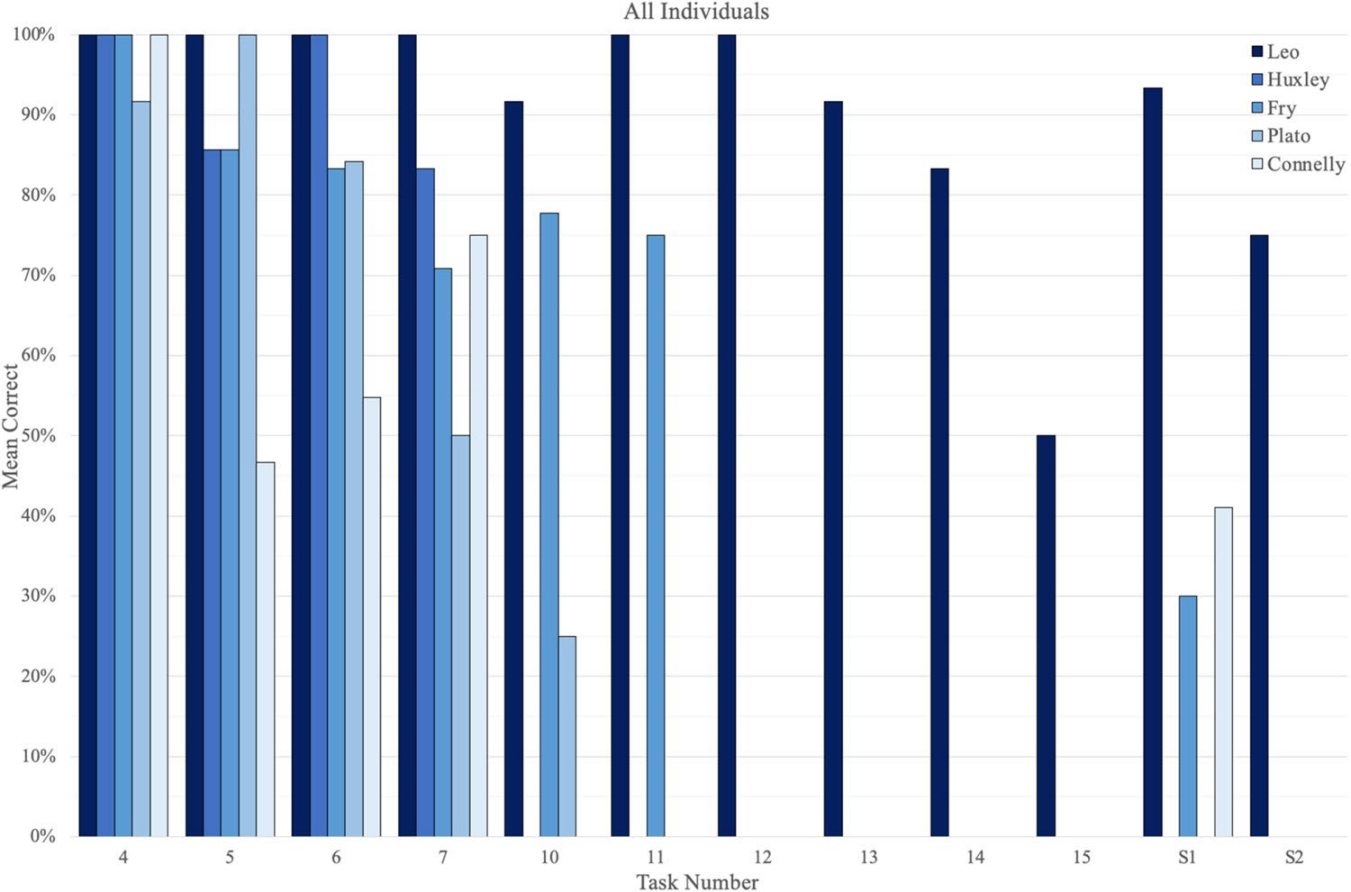
Results for all individuals, in percentage correct, for Tasks 4–8, 10–15, and S(1 and 2 swaps). Task 16 is excluded as it could not be coded into “percentage correct.” When applicable, percentages are averages of all rounds received by a bird. (Color figure online)

Rooks in the compartment saw manipulations on a wooden board, ~115 × 60 cm, placed at experimenter’s chest height (when standing on a footstool; see Supplemental Fig. 1A). Rooks tested in the aviary stood on a transparent plastic box placed against one side of the aviary, ~80 × 80 × 50 cm (see Supplemental Fig. 1B). Occluders consisted of small black plastic cups (~6.5 cm diameter, ~4 cm depth; see Fig. 3A) for Tasks 4–8 and S, and upright cardboard screens (~20 × 17 cm; see Fig. 3B) for Tasks 10–16, with cups when necessary. For Tasks 11 and 12, a cardboard barrier was placed vertically, oriented perpendicularly to the two test barriers and between them, so a bird had to choose a side to check, and could not see the other side when incorrect (see Fig. 3B). For Tasks 13–16, the upright cardboard barriers were instead fitted with cardboard flaps on each side, so the bird had to peer behind each barrier to check it, rather than seeing behind all barriers at once. Both were intended to clarify which blind the bird was checking when making a choice (see Fig. 4).

**Fig. 5:**
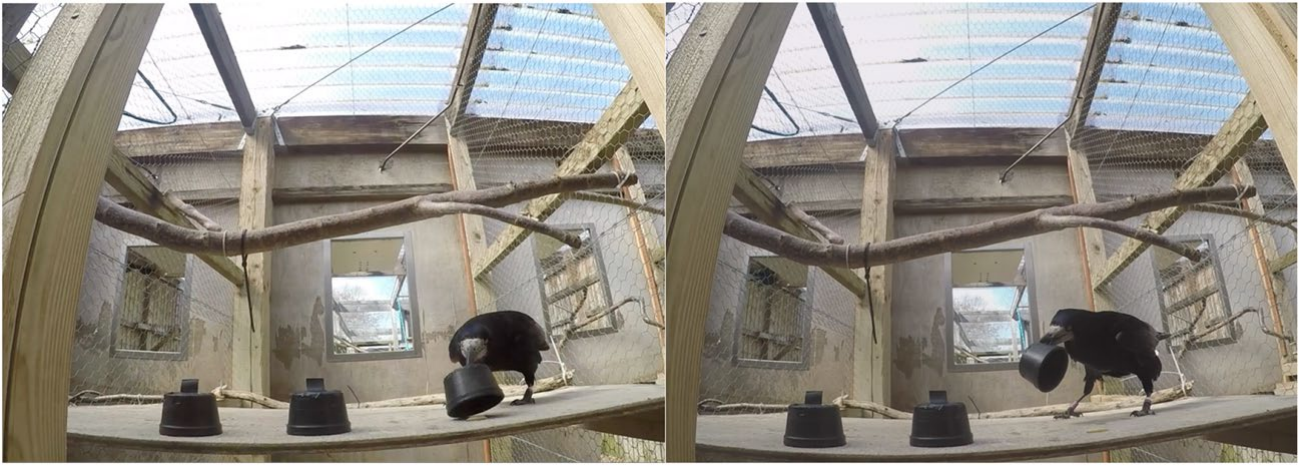
Leo demonstrating a successful “lifting” of the cup, by using the electric tape lip (and successfully finding the worm)

Cups had a central, small “hold” of folded electrical tape, so rooks could lift them with the beak (see Figs. 3A and 5). For tasks in which multiple occluders were present, cups or screens were individually marked with a dot of red, yellow, or light blue paint (~2 cm diameter; see Fig. 3A–B); marked covers have also been used for parrots, children, and great apes (Bremmer, 1978a, 1978b; Butterworth et al., 1982; Goldfield & Dickerson, 1981; Okamoto-Barth & Call, 2008; Pepperberg & Kozak, 1986; Pepperberg et al., 1997). Additionally, Jaakkola (2014) pointed out that supplemental videos of an OP study with Eurasian jays also show variation in the blinds (Zucca et al., 2007). Finally, this more closely matched the format of planned reasoning by exclusion tasks for which this experiment was a precursor (Pepperberg et al., 2013, 2019). Occluders were not varied (e.g., using cloths, boxes) to avoid unnecessary fear responses requiring extended habituation in these very neophobic rooks. Likewise, hidden objects were always edible (live waxworms for Tasks 4–8, 11–15, and S; live mealworms or peanuts for Task 16). All occluders were stored with the food each night, so that any odor traces were unlikely to be useful. Additionally, one task was an olfactory control, to ensure birds could not smell rewards. All trials were recorded with a GoPro Hero4 video camera strapped to F.M.C.’s chest. She wore opaque, nonreflective sunglasses in tasks with multiple options (Tasks 5+) to avoid gaze-cueing. Additionally, when possible (when it would not scare a bird already choosing), she would touch all or some of the other covers randomly, so that the last cover touched by her hand was not a reliable cue (full descriptions of all task setups in the Supplementary Materials).

### General procedure

**Fig. 6:**
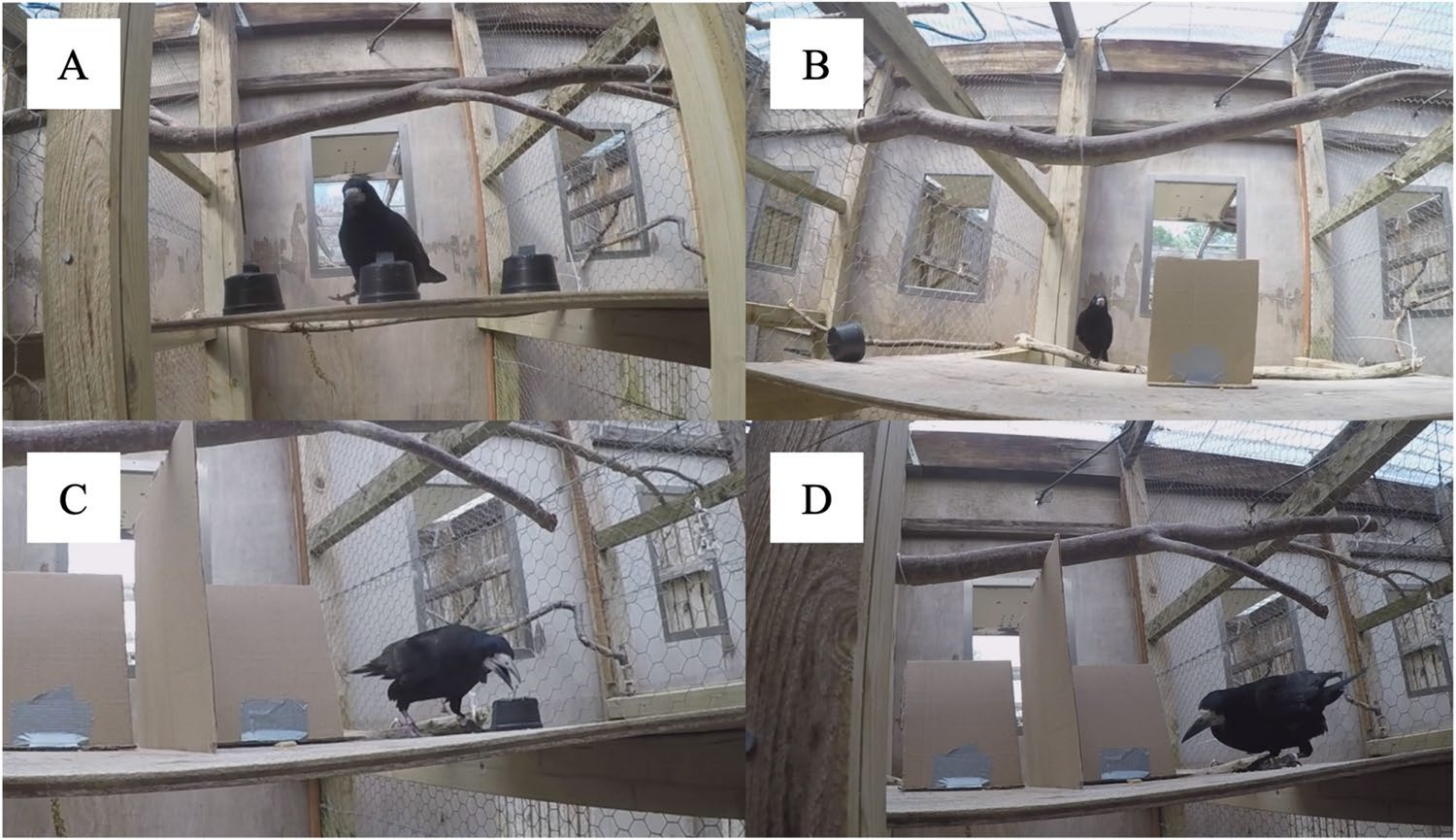
**A** Leo approaching a cup during Task 8, from experimenter perspective. **B** Fry being presented with Task 10, from experimenter perspective. **C** Leo opening a cup not shown to be empty during a Task 11 control (note worm behind barrier to experimenter’s right). **D** Leo obtaining a worm during Task 11, from experimenter perspective

**Fig. 7:**
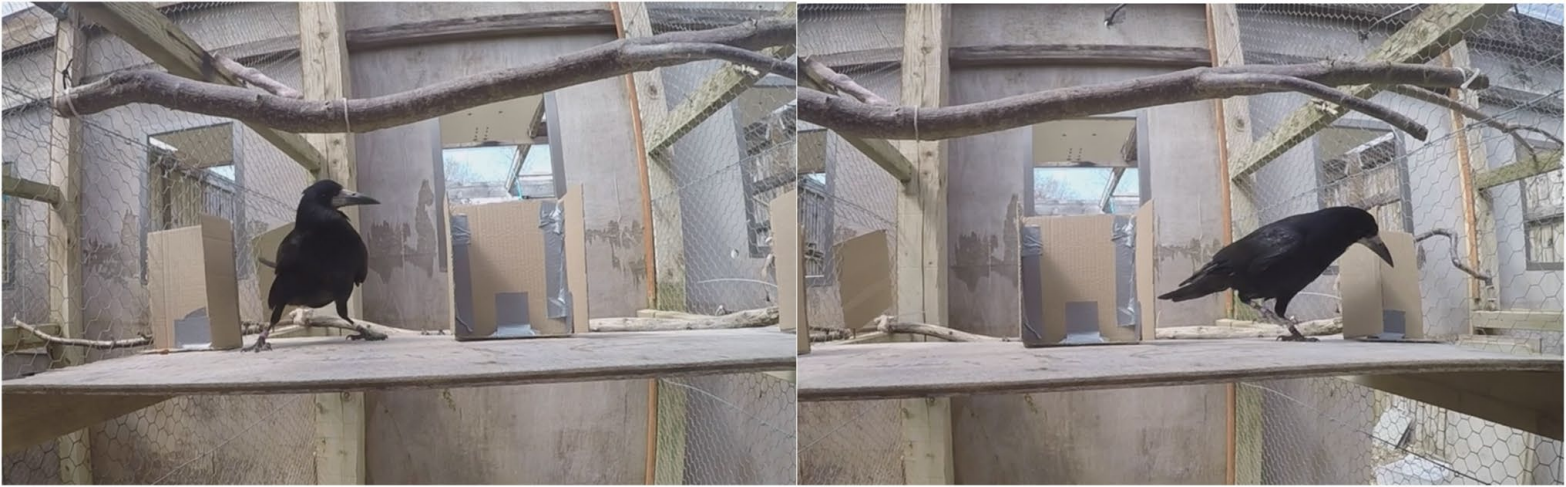
Task 16—From left to right, Leo abandoning the unexpected peanut behind the barrier to experimenter’s left, and approaching the barrier to experimenter’s right to check, possibly for the expected waxworm

**Fig. 8:**
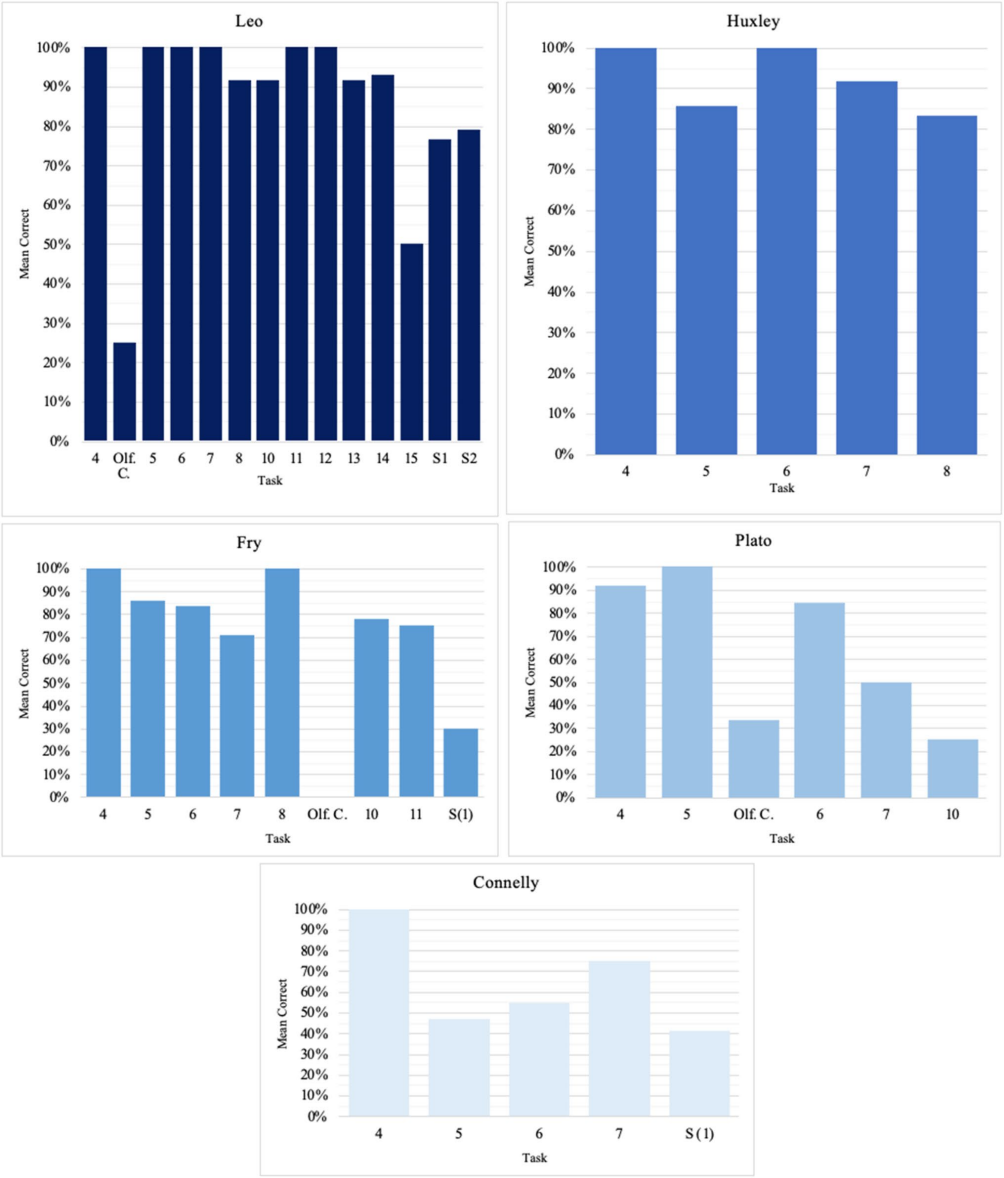
Results for each individual, in percentage correct, for Tasks 4–8, 10–15, and S(1 and 2 swaps). Task 16 is excluded as it could not be coded into “percentage correct.” When applicable, percentages are averages of all the rounds received

Rooks were presented with tasks from Uzgiris and Hunt’s (1975) Scale 1, beginning with Task 4 (complete occlusion); Task 9 was considered redundant and skipped, and Tasks 16 and S were added (e.g., Hoffmann et al., 2011; Pepperberg et al., 1997; see Zewald & Jacobs, 2021). Testing started with Task 4 because, during habituation to the experimenter, the testing compartments, and the first (single, unmarked) cup, all participating birds had either lifted cups to find hidden worms or watched other birds do so. However, no rooks had received Tasks 5+. Once rooks voluntarily entered the testing area, they were shown a waxworm. Then, the experimenter performed task manipulations, and randomly touched a few or all blinds: This was done as often as possible to prevent rooks from forming associations such as “choose the blind touched last.” Yet this depended on whether rooks were rushing forward or waiting patiently: To avoid decreasing their motivation by frightening them, this was considered sufficient, rather than scaring the bird to touch *all* the blinds every time (this was attempted but found to be very aversive). Finally, birds made a choice (see Figs. 6, 7 and 8). If incorrect, rooks were shown the absence of the reward but not where it was. Like touching all blinds after manipulations, it was highly aversive to shoo the bird away (usually, the bird would no longer return that day); however, when worms were removed after incorrect choices, they were not shown to the bird (i.e., the experimenter would lift only the back of the cup, collect the worm, and hide it in her hand, then lift the cup). If correct, rooks were allowed to collect the reward.

**Fig. 9:**
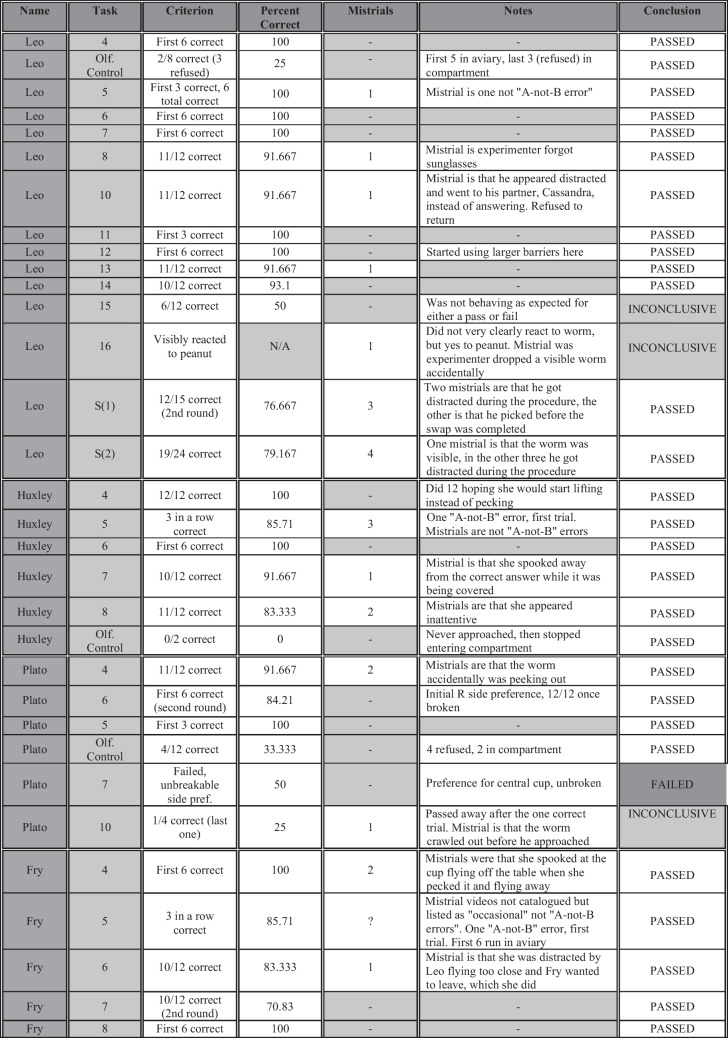

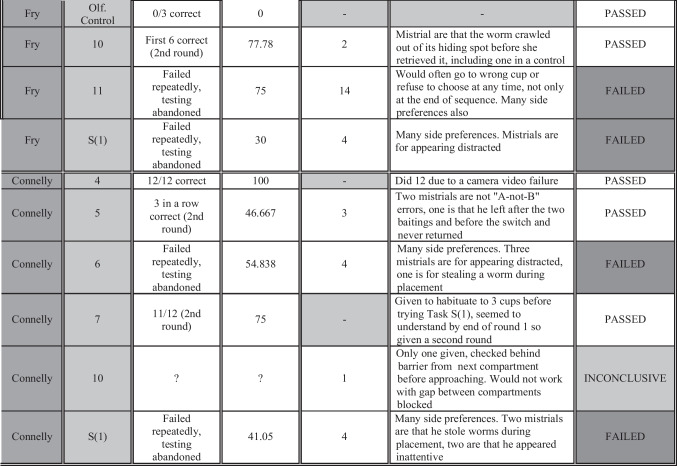
Additional information for all birds and tasks, including mistrials, percentage correct per bird per task (if more than one round was run, the number reported is the average of all), any notes, concluded outcome, and the criterion used to make the determination. Tasks are listed in the order each bird received them

If rooks left the compartment abruptly, the trial was considered “not attended” and repeated: This was judged a failure of motivation, or a fear reaction, rather than OP failure—rooks were often scared by sounds, called away by mates, or left to engage in other behaviors (e.g., eat, drink, bathe, food share). Exceptions are the Olfactory Control and Task 10, for which not searching by birds that usually would was considered indicative. Trials were not begun, or marked as mistrials if accidentally begun, if the rook did not appear to be paying attention. Behaviors that indicated the subject was not ready to begin the trial included: 1. Not looking at the setup or manipulations, such as looking around the compartment or into the aviary. 2. Being frightened. 3. Calling to experimenter or other rooks. 4. Preening, napping, regurgitating, or eating food. Additionally, mistrials could be declared due to experimenter error. Trials scored as mistrials were repeated directly (see Fig. 9). As part of an unrelated study (in preparation) with the same sample, conducted with nearly identical mistrial criteria, a blinded interobserver reliability measure was run: A student familiar with that study and with the mistrial criteria was given a set of 40 randomly selected videos, 20 of which had been classified as mistrials (consisting of 38.46% of mistrials at the time for that study) and 20 which had not. She was asked to decide blindly if a mistrial had occurred and, additionally, what the response behavior was, without knowing the main experiment’s judgment for each. Interobserver reliability was very high, with 18/20 videos of each category correctly assigned (Cohen’s kappa: 0.895). Additionally, the blinded observer classified all four trials that had been scored as correct also as correct, as well as an additional two trials which had been scored as incorrect.

Rooks were considered to “pass” a task if they fulfilled predetermined criteria (see the Supplementary Materials). These were relatively more stringent than in other studies for significance at the two-tailed binomial level (chance = 0.5; in most tasks here this criterion was 10/12 trials, or the first 6 in a row, correct). In other studies 5/6, 7/9, 5 in a row, or even 3 in a row correct would be considered passing (e.g., Bugnyar et al., 2007; Hoffmann et al., 2011; Salwiczek et al., 2009; Ujfalussy et al., 2013; Wang et al., 2021; Zucca et al., 2007). Additionally, because subjects may be tested until they either pass or fail repeatedly (e.g., Auersperg et al., 2014; Salwiczek et al., 2009; Zucca et al., 2007, among others), if a rook failed a task, it was allowed to try again. Each set of 12 trials given for a task constituted one “round” of trials, with the number of rounds taken to pass being noted when a rook did not pass a task immediately. Rooks should either continue to fail, or fail successive tasks, if they truly do not have the competencies required (but passing later tasks presupposes competence on earlier tasks; e.g., Pepperberg & Funk, 1990; Pepperberg & Kozak, 1986). If rooks exhibited neophobia to new tasks, they were fully habituated to the setup before continuing (details, descriptions, and photos of each task are in the Supplementary Materials, but see conceptual descriptions, and the OP stages associated with them, in Fig. 2). Methods for this study were preregistered on the Open Science Framework prior to the start of testing, but methods described here are as ultimately conducted.

## Results

### Olfactory control

No bird could find hidden worms without being given information as to their hiding place: Leo, 2/8 correct (last 3 refused); Plato, 4/12 correct (2 refused); Huxley refused to choose in both trials received and then began to be hesitant to return to the compartment, so olfactory control testing was discontinued; Fry refused to choose in 2/3 trials and chose incorrectly in the one she attempted. Connelly did not receive olfactory controls because his at-chance performance on Tasks 6 and S suggest he could not find the reward.

*Task 4—Finding an object that is completely covered:* Leo, Fry, Connelly, and Huxley passed with the first 6 correct, removing cups to retrieve fully hidden worms; Plato with 11/12.

*Task 5—Finding an object completely covered in two places:* All rooks that made “A-not-B” errors by persisting to search in an incorrect, previously rewarded location eventually ceased. Leo and Plato never made any (Leo, 6 trials; Plato, 3 trials^1^). Fry and Huxley made one “A-not-B” error in their first trial, then chose correctly 6 times in a row. Connelly made 7/15 “A-not-B” errors (Trials 1–4, 6, 9–10), making no “A-not-B” errors on the last 5 (first half, 2/7 correct; second half, 5/7). Rooks rarely made errors that were not “A-not-B errors,” erring in either of the trials before the switch: These trials were counted as mistrials as they were not considered to be testing the concepts of Task 5.

*Task 6—Finding an object completely covered in two places alternately:* Leo and Huxley passed with the first 6 in a row correct, removing cups to retrieve fully hidden rewards in alternating locations; Fry with 10/12 correct. Plato failed his first round (9/12) due to a clear right-side preference (see the Supplementary Materials—Additional Notes), but was 7/7 correct on his second round once it was broken intentionally. Connelly received 10 rounds (12 trials each) of Task 6 plus 4 additional trials, but developed frequent side preferences that had to be broken repeatedly, and never passed (54.84% correct in total). Connelly showed no evidence of learning (35/62 correct in the first half, 33/62 in the second, Fisher’s exact test, two-tailed).

*Task 7—Finding an object completely covered in three places:* Leo passed with the first 6 correct, removing cups to find fully hidden worms in three possible locations; Huxley with 11/12 correct. Fry failed her first round, during breeding season, with 7/12 correct, then passed her second round (right after breeding season) with 10/12 correct. Plato failed his first round with 6/12 correct, then developed an unfixable preference for the central cup. Connelly was given Task 7 to habituate him to the presence of three cups before being moved on to Task S(1); he failed his first round (7/12 correct) but passed his second with 11/12 correct.

*Task 8—Finding an object after successive visible displacements:* Fry passed with the first 6 correct, by tracking a worm placed under all three possible hiding places sequentially to its final location; Leo with 11/12 and Huxley with 10/12 correct (see Fig. 9). Given their performance, Plato and Connelly were not offered Task 8.

*Task 10—Finding an object following one invisible displacement:* Leo passed with 11/12 correct, searching for the worm behind a barrier that the occluding cup had passed behind after seeing the cup was empty. He received two controls: In one, the worm was not left behind the barrier but shown to be under the cup after manipulations (Leo ignored the barrier and correctly went to the cup); in the second, the worm was left behind the blind but the cup was not shown as empty (Leo correctly checked the cup and then the blind). Initially, Leo would check behind the barrier from the side by going to next compartment, so 12 trials were conducted: He ceased by the end of the set and his path was subsequently blocked by cardboard.

Fry passed with 6/6 correct on her second round (8/12 on the first, by not searching in the first four; see Fig. 9). She received 4 controls in which the worm was left behind the barrier, but the empty cup was not shown: in all (except for one mistrial, in which the worm crawled out from behind the barrier) she went to the cup first, then looked behind the barrier. Likewise, she received 4 controls in which the worm was left in the cup and the cup was not lifted: here too she always went for the cup. Connelly was moved on to Task 10 after constantly developing side preferences on Task 6: he correctly retrieved the worm from behind the barrier on his first trial but not before going to the next compartment to look behind the barrier from the side. Blocking this path with cardboard did not work because he refused to enter the testing compartment at all. Plato was moved on to Task 10 due to his unbreakable central cup preference in Task 7; he did not approach on three trials then got one trial correct before dying of natural causes before further testing. Huxley would no longer remain in the compartment long enough after Plato’s death, so she did not receive testing after Task 8.

*Task 11—Finding an object following one invisible displacement with two screens:* Leo passed with the first 3 correct (see Fig. 9), searching behind the correct screen visited by the occluding cup rather than persisting in choosing a previously rewarded, incorrect location. Leo received one control: The worm was not left behind a barrier, and the cup was not lifted, to ensure Leo had not just learned a strategy “go to cardboard visited” (he checked the cup instead of going to the barrier). Fry eventually accumulated 3 correct sequences and one “A-not-B” error, but made a very high number of not “A-not-B” errors and developed strong, difficult-to-break side preferences, particularly for the left blind, which she had not done previously. Fry showed no evidence of learning (2/9 correct in the first half, 1/9 correct in the second; Fisher’s exact test, *p* = 1, two-tailed).

*Task 12—Finding an object following one invisible displacement with two screens alternated:* Leo passed with the first 6 correct, finding worms behind alternating barriers once the intermediate occluding cup was shown to be empty. Leo also received controls—one as in Task 11, in which he went for the cup, and two in which the barrier was baited, but the empty cup was not shown. On a mistrial, he visibly noticed the worm behind the barrier as he approached the cup; on the second try, he opened the cup first and went to the barrier second.

*Task 13—Finding an object following one invisible displacement with three screens:* Leo passed with 11/12 correct, finding worms behind three possible barriers after seeing the intermediate occluding cup was empty. Leo received three controls in which a barrier was baited but the cup was not shown; in one, he went for the barrier directly for the first time.

*Task 14—Finding an object following a series of invisible displacements:* Leo passed with 10/12 correct, finding worms in logical hiding places once the intermediate occluding cup visited multiple screens and was shown to be empty; he went directly to the third (rewarded) screen on 8 trials, including the first; went to the first screen then kept searching on 2 (showing persistence that is counted as correct); and went to the first screen then did not search further on 2 trials (errors). In the drop-first controls, he behaved appropriately in 2/5 trials: He did not seem to understand that being shown the empty hand meant the worm was already dropped, and he continued to pick the third or first blind even when the worm had been dropped in the second. However, this also shows that he could not see or smell the worm throughout, as he did not correctly find it here. Leo was given 12 more trials of Task 14 to see if he would switch to going to the third blind first consistently: He still went to the first blind first on 4 of these, but went to the third blind first 8 times in a row. He was given 4 “drop first” controls and passed 2 of them. Considering his behavior was not illogical (except for in the controls, the worm could be in any blind, and he only ceased searching twice), he was moved on to Task 15.

*Task 15—Finding an object following a series of invisible displacements with evidence of representation:* Leo behaved unexpectedly, neither consistently choosing the first or the last screen when he should have learned from the previous task that the worm was always placed under the last screen: instead he went to the first screen first on 5/12 (including his first), and to the third first on 7/12 trials (on one, he did not search further). He received two controls in which blinds were baited surreptitiously: He never found any worm in this way.

*Task 16—Finding an object following a series of invisible displacements with evidence of representation by substitution:* Leo showed surprise at the reward being surreptitiously substituted by behaving differently than previously. On the first 6, a mealworm was used. Leo took it, and though it seemed he hesitated slightly on the second trial, and he checked both the first and third blinds on Trials 3 and 4 (after having found the worm), this was only a very subtle impression by the experimenter rather than very conclusive behavioral signs of violation of expectation. He received two more using a peanut: In the first, Leo clearly reacted—he examined it, left to check the third blind, came back, examined the peanut again, left to the perch, waited, examined it again, went to the hatch, waited almost a minute, checked the peanut again, then left to the perch again (see Fig. 7). The experimenter did not think he would take a peanut, so she terminated the trial. However, on the next trial he hesitated but took the peanut.

*Task S(1)—Shell Game with one swap:* Leo initially failed Task S(1), in which he was supposed to track the final location of a worm hidden under a cup after this was swapped with one empty cup, with 11/15 trials correct and 2/3 controls correct (the baited cup does not move, and only empty cups are swapped),. After the first 9 trials (in which he made 4 of his 5 errors, including the control), he refused to participate for almost 2 weeks, during breeding season. After, he made one error on the rest of the set. On his second round, he passed with 12/15 correct, and 2/3 controls correct. Two additional controls, ran subsequently the same day, were both correct. It was decided to move Fry and Connelly to Task S(1) from tasks at which they stalled; Fry, because she seemed averse to the vertical blinds for Tasks 10–16, and Connelly, because it was hypothesized the cup movements might force him to focus and prevent side preferences. Fry received 30 trials, being correct on 9 of them (30%); she was correct on only 1/8 controls and became increasingly reluctant, so testing was terminated. Connelly, who was more willing to work, received 95 trials, and was correct on 39 of them (41.05%, controls included; he was correct on 9/21 controls, 42.857%), before testing was terminated. Neither Connelly nor Fry showed evidence of learning (Fry, 6/15 correct in the first half, 3/15 in the second, Fisher’s exact test, *p* = .427; Connelly, 22/47 in the first half, 17/48 in the second, *p* = .3006, both two-tailed).

*Task S(2)—Shell Game with two swaps:* Leo received an additional set with two swaps—either both, one, or none involving the baited cup. Out of 24 trials, he was correct on 19.

## Discussion

The rooks tested in this series of experiments show strong object permanence abilities. The only rook to complete the entire set of tasks presented, Leo, showed full Piagetian Stage 6b OP; his performance and the conclusions drawn from it will be discussed in the next section. Other rooks differed in performance, with two rooks performing well who could not continue testing due to external circumstances (Plato, by his death; Huxley, by being unable to remain in the testing space without Plato warding off other birds). Huxley, Fry, and Connelly demonstrated full or partial Piagetian Stage 5 object permanence: Huxley and Fry by completing tasks up to Task 8, demonstrating Stage 5b, and Connelly by ultimately passing Task 7, demonstrating Stage 5a. Plato may have also done so, if his singular correct trial on Task 10 before death is indicative, which would correspond to Stage 6a; however, his death renders this inconclusive, and he can only be firmly assumed to have demonstrated Stage 5a by passing Task 6. Fry also demonstrated Stage 6a by passing Task 10. Both Fry and Connelly were unable to pass Task S during this study, however, so it cannot be concluded that they demonstrated Stage 6b. Potential reasons for individual differences will be discussed below.

### Leo

Leo conclusively demonstrated Stage 6a by passing all tasks up to 13 in one round; he also demonstrated Stage 6b by passing Tasks 14 and S(2) in one, and Task S(1) in two, rounds. He made no “A-not-B” errors in Tasks 5 and 11. His unusual behavior in Task 15 is of interest: He did not appear to learn to always go to the final barrier visited by the occluding cup on Task 14, when it was the only rewarded barrier in every non-control trial, despite being given additional trials, and accordingly chose the first barrier visited by the occluding cup almost half the time in Task 15, including on his first attempt. It is clear he could not perceive the worm behind barriers: in addition to the aforementioned controls (see the Results section), he should instead have always chosen the baited barrier first in Tasks 14–15 if so. It is possible that the additional controls (which were implemented during the procedure rather than post hoc specifically to ensure he could not see) might have suggested to him that experimenter behavior during manipulations was not indicative and, as he was allowed to search in these tasks, a reasonable course of action would be to choose any barrier and continue searching.

Leo’s behavior is in line with those of carrion crows and ravens, many individuals of which also fail Task 15: the crows also never mastered transpositions (similar to Leo’s Task S(2), but with the target always moving once in the two swaps), and made “A-not-B” errors, as did the ravens, yet researchers concluded they mastered full OP (Bugnyar et al., 2007; Hoffmann et al., 2011). Magpies have also been argued to have strong or full OP despite failing Task 15 (although in their case, and in the case of the crows, most failed to make extensive searches, unlike Leo who failed to search on only one occasion; Hoffmann et al., 2011; Pollok et al., 2000). Some individuals of azure-winged magpies and jackdaws also fail Task 15, whereas other pass (Ujfalussy et al., 2013; Wang et al., 2021); Eurasian jays are reported to pass Task 15 (Zucca et al., 2007). Additionally, Leo’s behavior in Tasks 14 and 15, while initially unexpected, is not unusual in corvids. In addition to individuals of crows, ravens, and azure-winged magpies, Eurasian magpies also failed Task 15 and two individuals were reported to check the first blind first on occasion (Pollok et al., 2000). Jackdaws are reported to pass Task 15 by searching in reverse order of hiding, but it is noted that five individuals continued to search in the first location of hiding, as Leo occasionally did: It was concluded these behaviors did not justify a conclusion of inability to display full OP, because it remains a valid way of solving the task (Ujfalussy et al., 2013).

Indeed the methodology for Tasks 14 and 15 appears to vary between bird studies. Some explicitly indicate in their methods that the target object was always left behind the final barrier visited in Task 14, and transfer directions switched, as conducted in this study and in a number of previous studies (e.g., Bugnyar et al., 2007; Hoffmann et al., 2011; Pepperberg & Funk, 1990; Pepperberg & Kozak, 1986; Pollok et al., 2000; Ujfalussy et al., 2013; Zucca et al., 2007), while others are less clear (Funk, 1996; Wang et al., 2021). In at least two the final location of the object was not always behind the third barrier visited in the transfer sequence, but varied depending on when hands or occluders were shown to be empty (akin to some of the controls in this study; Auersperg et al., 2014; Pepperberg et al., 1997), thus requiring different responses for each trial in accordance with the logical possibilities presented. Note that Uzgiris and Hunt’s original 1975 Scale 1 specifies the order of transfer must *always* be in the same direction in all trials of both Tasks 14 and 15 (pp. 163). However, in corvid and parrot studies, when it was possible to identify whether the direction of transfer was always kept constant or could start on one side and then the other, it always varied (the transfer order could either be ABC or CBA, if barriers are labelled as A, B, and C from one side to the other; Hoffmann et al., 2011; Pepperberg & Funk, 1990; Pepperberg & Kozak, 1986; Pollok et al., 2000; Ujfalussy et al., 2013; Zucca et al., 2007; this study. NB: In the case of Hoffmann et al., 2011, this determination is assumed from descriptions including “the bird had to search for the worm directly behind the last screen or behind all three screens in the same order as the experimenter’s hand passed behind them,” pp. 362, rather than indicating there was a first, second, and third screen; a similar determination was made in the case of the Wang et al., 2021; and for Pepperberg & Funk, 1990, for closely following the method of Pepperberg & Kozak, 1986, where this is clearly stated).

This discrepancy is problematic not only because it makes interspecies comparisons difficult, but because the most common method of applying the task (always to the third barrier visited in a linear sequence, and starting from both sides in different trials, as used here), is difficult to interpret when unexpected, but logical, responses occur. In an OP study examining chimpanzees, orangutans, and children, searching multiple occluders was intended to indicate that a subject is able to mentally reconstruct the path of the target, but it is acknowledged that such tasks could also result in a strategy of searching under all occluders (Call, 2001). Although it is expected that subjects would have learned from Task 8, which is Task 14’s visible displacement counterpart, that the reward is always left at the last barrier to which it is transferred, such learning might not always occur (NB: Uzgiris & Hunt’s original Task 8 specifies the transfer can be in any direction for Task 8, but must *always be in the same direction for Tasks 14 and 15* because demonstrating that subjects can reconstruct the path of the target object is of specific interest, and can only be assumed from visits to the second barrier in the sequence when searching in order, even though it is never rewarded; Uzgiris & Hunt, 1975). Task 8 can be solved by searching the last place the target object was seen, a cue that is not available in Tasks 10–15 once the intermediate cover is shown to be empty. Although Task 14 in part requires that subjects infer the object is left at the most recently visited barrier once the intermediate cover is shown to be empty, this inference may not be made reliably, and since searching is allowed, there is little cost in failing to make it. Additionally, subjects might not have paid attention to the rule regarding the final location of the object in Task 8 (always the third barrier visited, in either direction) and may be attempting to learn it from scratch in Task 14, usually in a short number of trials, since searching in reverse order is still considered correct.

Additionally, although both “unsolvable” controls, intended to determine that the subject cannot sense the reward, and “drop-first” and “drop-last” controls, intended to exclude the use of local rules, are of extreme importance in these tasks, they may add to the subjects’ confusion about task affordances. For example, if the subject has experienced such “tricks” before or during the application of Task 14, as was the case here, and had failed to learn the “search under the last barrier” rule in Task 8, as Leo might have failed to do, it is indeed possible that the subject might conclude that any barrier could possibly be baited in any given trial of Tasks 14 and 15. A reasonable course of action then might be to pick any barrier to inspect first, according to any number of parameters (proximity, side preferences, local events around the bird such as noises, etc.) and to continue searching if incorrect.

If, from the perspective of the bird, the reward could be anywhere, the only logical way to “fail” Tasks 14 and 15 from purely an OP perspective is to stop searching, which Leo almost never did. Despite the scale criteria requiring subjects to learn to visit the last barrier in Task 14, and then to continue this proclivity in Task 15 but continue searching when incorrect (with this being the key behavior which demonstrates evidence of representation), this becomes of little meaning when animals do not learn to visit the last barrier only in Task 14 (a logical option here, from an OP perspective, since the target object *could* theoretically be anywhere when critical controls have been used, and extensive searching is always allowed). Learning “the object is always under the last barrier visited, in any direction, except for occasionally in controls” is more difficult, and not the same, as learning “the object is always under the barrier on the left” (as Uzgiris & Hunt’s original method would allow for). Additionally, it is possible that animals that *do* learn to search under the last barrier visited in Task 14 might then continue it through strong perseverance, engendering failures in Task 15. Consequently, the results observed in the corvid family with regard to Tasks 14 and 15 are more likely to failures of rapid learning, working memory, or even reasoning by exclusion, rather than failures of OP.

The less commonly used method of presenting these tasks, in which responses change depending on what the animal is shown, may be more fruitful for future study (Auersperg et al., 2014; Pepperberg et al., 1997). For instance, a “revised” set of Tasks 14 and 15 could either return to the original Uzgiris and Hunt (1975) method in one direction only, but include appropriate drop-first controls in which the subject should have only one logical option for where the reward should be. Alternatively, the direction of baiting can continue to vary, but researchers must think very carefully about the timing of controls: Including them throughout the study, as was done here, might suggest to the subject that experimental manipulations are not always reliable, and that picking any barrier and continuing to search is a worthwhile rule. On the other hand, including them only at the end of Task 15 might be too late if subjects have learned a strong “go to the last barrier visited” rule, as some of the corvids who failed to search further on Task 15 may have done (e.g., Hoffmann et al., 2011; Pollok et al., 2000). Ultimately, well-implemented tasks like Task S and translocations (not used here) may be more informative for assessing multiple invisible displacements.

Leo’s behavior in Task S(2) (79.167% correct) indicates some ability to follow multiple invisible displacements. Although most of his errors occurred when the baited cup was indeed swapped twice (the most difficult type of trial; he was 3/6 correct), he could still sometimes follow these very difficult displacements, and was not fooled when two swaps occurred but the target object was swapped once (10/12), or when two empty cups were swapped (6/6 correct). Here the use of marked covers may have helped Leo somewhat: Okamoto-Barth and Call (2008) analyzed the effect of using marked vs. unmarked covers in children and great apes and concluded that differently marked covers did increase their performance after invisibly conducted rotations, but only for the older human children. However, Jaakkola (2014) pointed out this may have made the rotation tasks more akin to visual displacements, but that study dealt with invisible rotations, rather than transpositions, so different cognitive mechanisms may apply. Additionally, only the older children benefited from this cue.

Most OP studies in birds do not test for Task 16 or S: When they do, procedures vary, and in most cases only one switch of the baited cup is examined, whereas Leo encountered up to two (Hoffmann et al., 2011; Pepperberg et al., 1997; Ujfalussy et al., 2013; Zucca et al., 2007). Despite Task S(1) being cognitively demanding, it may be testing for OP Stage 6a (single invisible displacement) instead of 6b (multiple invisible displacements), as the target object is transferred once. Leo’s Task S(2), in which the target object could be swapped twice, logically targets OP Stage 6b: One study with Goffin’s cockatoos also included a condition with two swaps of the target, which most cockatoos passed by their second attempt (Auersperg et al., 2014).

Likewise, although interpreting signs of “surprise,” “frustration,” or violation of expectations in animals is difficult, Leo’s behavior in one trial of Task 16 is particularly compelling. When the expected waxworm was secretly substituted with a mealworm, he appeared qualitatively to hesitate slightly, and checked additional blinds, but took the worms; however, in his first trial in which a waxworm was substituted with a peanut, Leo clearly reacted to the switch (see video in the online Supplementary Materials). He initially did not take the peanut, checked another blind, waited, checked the peanut more times, and continued to behave oddly as opposed to his usual retrieval of the reward. In no normal instance was Leo observed to behave in this way, and it is suggestive that he noticed the reward was not as expected. Having an expectation as to what the reward was may be evidence of representation of the reward even once it stops being visible (Pepperberg et al., 1997).

Additionally, there is no evidence that olfactory cues, or subconscious cueing, are to account for the successful performances in this study, given the anti-cueing measures and controls throughout. The rooks’ performance is also unlikely to be accounted for by rapid learning, despite the tasks being presented sequentially: Leo almost always passed tasks in his first round, and often made correct choices from the first trial. Likewise, when rooks struggled with tasks, there was no evidence of learning: Fry and Connelly were never able to pass some tasks despite extensive experience—Tasks 11 and S(1) for Fry, 6 and S(1) for Connelly. They also did not make significantly more correct choices in the second half of trials (see the Results section). Learning during OP tasks was examined but also not observed in azure-winged magpies (Wang et al., 2021).

Although only Leo received and passed the full set of OP tasks presented, an argument can be made for his performance being indicative of rooks. Object permanence is not considered to be an ability present in only a few individuals of a species; most studies of OP in which individual performances vary, or in which some drop out or were excluded from testing, have extended the competence of the most advanced individual(s) tested to the likely competence of the species (see Pepperberg & Funk, 1990; Pollok et al., 2000; Salwiczek et al., 2009; Triana & Pasnak, 1981; Ujfalussy et al., 2013). As elsewhere in comparative cognition, power studies (in-depth studies completed satisfactorily and with well-controlled methodologies by only one or a few individuals) are still thought to be likely to represent the maximum capacity of the species when the individuals studied display passing performance, although the capacity may not be evident or expressed in all individuals, and the failure of others cannot invalidate this success (Pepperberg & Funk, 1990; Pepperberg & Kozak, 1986; for a discussion, see Triana & Pasnak, 1981).

Leo is also likely to fit the proposed “STRANGE” framework of research subjects in comparative cognition (Webster & Rutz, 2020). Although all rooks were socially housed as a group, hand-raised similarly, and were wild-caught as nestlings from the same area around the lab (although the genetic relationships between them are mostly unknown, Leo and Cassandra were from the same nest), Leo was prone to self-selection in participation in this and subsequent studies (more in Neophobia, below). He was among the first to engage with the experimenter, testing space, and materials (see Neophobia), regularly came in for testing many more times per day than any other bird (F.M.C., personal observations), had relatively little to no experience with previous physical cognition experiments, and appeared particularly motivated to receive high numbers of food rewards per session and day, as well as being dominant enough to flush other birds away from the testing space (see Dominance, below). Leo might have been particularly primed to succeed in these tasks, at an accelerated rate, rather than being cognitively superior to others. In addition to OP capacity, OP studies are likely to require other cognitive components, including motor skills, motivation, tractability or socialization, memory, attention (Pepperberg & Funk, 1990), and even secondary representation or inference (Jaakkola, 2014; Suddendorf & Whiten, 2001). Some factors that may underlie individual differences in OP performance here are examined and discussed below.

### “A-not-B” errors

The significance of initial “A-not-B” errors is not entirely understood—children, apes, parrots, ravens, carrion crows and azure-winged magpies generally make them, whereas magpies, Eurasian jays, and most jackdaws do not. The distribution of the occurrence of “A-not-B” errors within the caching and noncaching corvids tested does not fit the hypothesis that food storing behavior alone is to account for the lack of “A-not-B” errors noted in Eurasian jays and magpies, cachers, and the low levels noted in jackdaws (see Wang et al., 2021), noncachers, whereas crows and ravens cache and make these errors.

Given the low amount of “A-not-B” errors here (Leo and Plato, never; Huxley and Fry, once; Connelly, several), the executive function explanation may be correct: although not generally considered highly specialized cachers, rooks do cache in large quantities in the wild, and appear to accurately recover their stores after months (Källander, 2007). Likewise, developmental maturation of any system, or of OP competence, would not account for the “A-not-B” errors observed, and their quick cessation in most cases, since these rooks were all adults. It is proposed that the executive function explanation may hold the most explanatory power, and that rooks that initially made “A-not-B” errors may have had to exert additional executive control over their actions to cease making them. The use of marked covers may have aided the rooks’ performance with “A-not-B” errors: However, this did not entirely eliminate them in parrots (Pepperberg & Kozak, 1986; Pepperberg et al., 1997), and individuals corvids have been known to avoid “A-not-B” errors even with identical covers, such as magpies and Eurasian jays (Pollok et al., 2000; Zucca et al., 2007), and, to some extent, jackdaws, for which only 2/8 individuals made them (but the task required 3 repetitions before switching; Ujfalussy et al., 2013).

### Neophobia

The relative “boldness” of individual rooks has affected their behavior in cognitive tasks. In a study testing the effects of boldness (and pair bonds and dominance) on strategies in a string-pulling task, it was observed that “boldness” was a highly-repeatable metric in rooks, and that bolder individuals were more likely to “scrounge” under the apparatus than to attempt to “produce” by pulling strings (Jolles et al., 2013). Unfortunately, the names of birds participating in that study were not published, but it was the same sample. It is plausible that the bolder birds in the present study may have been more willing to participate here and to pay attention to manipulations, as retrieving the reward may have been akin to “stealing” from the experimenter, something less-bold birds may have been hesitant to do.

**Fig. 10:**
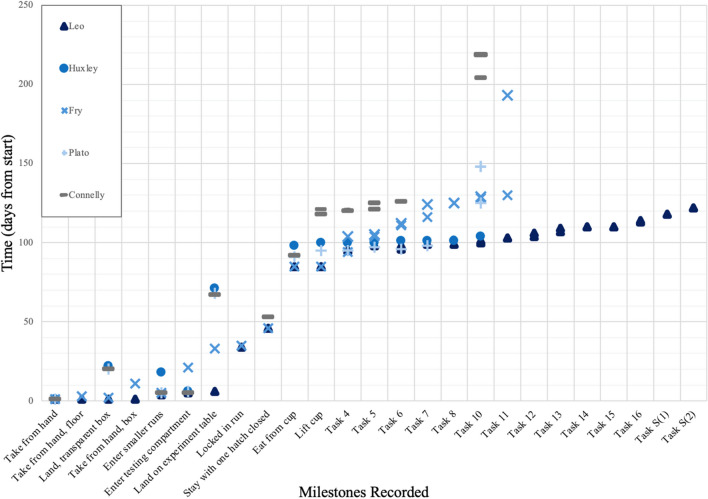
Plot of day of attainment, per bird, of each milestone recorded during habituation and testing. When two points are entered per bird for the same milestone, it indicates the day a task was begun and was completed or abandoned, when applicable. Task S(1) is not entered for Fry or Connelly, as the timing of its administration was decided by the experimenter, and milestones are ordered by logical difficulty level. NB: Locking runs/partially shutting hatches was abandoned because only Leo and Fry would tolerate it, and only briefly (minutes)

Although neophobia levels in the present sample were not directly assessed for this study, qualitatively individual differences in willingness to engage with the experimenter, the testing space, and materials was recorded (see Fig. 10). Leo was generally more likely to approach and touch a novel object or situation first, although Fry and Plato were also quite bold. Plato began working before Leo, but Leo quickly overtook Plato and was especially quick to habituate to increasingly complex testing setups (like additional cups, new barriers). Fry was very averse to the second barrier added for Task 12 (see Fig. 10). Connelly was the most hesitant with novelty, followed only by Huxley. Huxley was slow to start but progressed quickly until the first barrier was introduced, which she found aversive, and was not able to overcome this neophobia until Plato’s death. The barriers in fact seemed to pose the most problems in terms of neophobia and aversion to approach: not only did Fry take about 60 testing days to habituate to the second barrier in Task 12 (NB: The birds were encouraged to approach the testing setup with visible worms placed around the barrier, when habituation was required in response to fear being displayed, not by being offered task trials); Plato also did not become accustomed to Task 10 for about 20 testing days, and Huxley did not habituate in the approximately 50 testing days between finishing Task 8 and Plato’s death.

Fry’s poor performance starting with Task 12 and Connelly’s with Task 6 might thus be partly due to neophobia towards increasingly complex setups, numbers of occluders, or manipulations beyond those they were willing or able to attend to. Once their threshold of neophobia was reached, they may have reverted to side biases or random choice to maximize their odds of getting a reward by chance, without paying attention to the increasingly complex task contingencies. However, post hoc tests examining the effect of bird ID on either date of attainment or date of completion of the milestones examined were not significant (see the Supplementary Material, Habituation Information), so it cannot be directly concluded in this study that individual differences in neophobia strongly affected the results observed. Yet neophobic responses, and tractability, have been found to affect performance in OP studies: hand-raised kakarikis generally passed tasks sooner and with less errors than parent-raised individuals (Funk, 1996); increasing neophobia was hypothesized to have affected jackdaws who took longer to pass (Ujfalussy et al., 2013); and Eurasian jays tested at a younger age (before the corvid neophobic stage) performed better, passing tasks earlier and with less sessions “not attended”; increasing numbers of vertical blinds were also noted to induce neophobic responses in the jays (Zucca et al., 2007).

### Dominance

Although dominance was not explicitly tested in this study, and as such no detailed data-driven hierarchy can be presented, F.M.C. spent about 100 days habituating the rooks to her presence and to the testing setup before this study; much of this was spent in the aviary with the sample. At the beginning of habituation, Plato was observationally identified as the most dominant, successfully displacing all others for waxworms. Leo and Connelly, the other males, were next (but without explicit testing at the time, it could not be determined which was more dominant, assuming a linear hierarchy as has been reported in the past for this sample of rooks; Jolles et al., 2013), followed by Fry and Huxley, who were likely to be displaced by any male interested in the rewards. By the start of the experiment, Leo was the most dominant bird observationally (see the Supplementary Material, Habituation Information).

Dominance status is likely to affect behavior on cognitive experiments. A study examining dominance (and boldness and pair bonds) on strategies and performance on a string-pulling task in this same sample of rooks found an effect of dominance: more dominant rooks were more likely to act as “producers” by pulling the strings, rather than “scroungers” by retrieving fallen food from below the setup, and were also more likely to be successful in their producing attempts (Jolles et al., 2013). This is likely because the cost associated with time spent pulling the string to retrieve the food was less detrimental to dominant birds (which may be less likely to be displaced during an attempt) than subordinate birds (which are more likely to be displaced). The importance of considering social factors and individual repeatable patterns of behavior when conducting cognitive tests was underlined (Jolles et al., 2013; unfortunately the individual names of birds involved and the full hierarchy resulting from that study are not published). Likewise, the relative dominance of focal birds to their observers has been demonstrated to be a factor in caching: California scrub-jays protect their caches when observed by both dominant and subordinate conspecifics, but engage in higher levels of recaching when observed by dominant birds (Dally et al., 2005a, 2005b, Dally, Clayton et al., 2006a, Dally, Emery et al. 2006b, 2010); and Eurasian jays have been shown to cache more frequently in ground locations and to recache more often when observed by subordinate birds, but suppressed caching, cached more away from the ground, and further away from observer birds when these were dominant (Shaw & Clayton, 2012).

It is possible dominance was also at play here. Leo, as the rising most dominant bird immediately preceding the study and throughout, may have perceived a lower cost of remaining in the compartment frequently, for long periods, and of paying attention to the manipulations to “produce” the most rewards. He was unlikely to be displaced from the testing area or to have his rewards stolen, and it may have been the best strategy available to him, to maximize his rewards, to work attentively for them. On the other hand, birds that were more likely to be displaced, including having rewards stolen by Leo, may have benefitted from a different strategy—paying attention while tasks are simpler and quicker, and produce rewards attentively then, but switch to a “scrounger-like” tactic of picking randomly or developing side preferences when the attentional or time costs of the tasks became too high. This may have contributed to Fry and Connelly’s decline in performance beginning with Stages 6a and 5b, respectively. Huxley was clearly affected by dominance factors: Without Plato’s interference, Huxley was unable to continue participating in this study or subsequent ones.

### Individual idiosyncrasies

It is common for animals to develop side preferences when multiple choices exist: It may be sufficient to obtain the reward some of the time, especially in difficult or aversive tasks, by selecting one side regardless of presentation. When they occur, side preferences are often intentionally corrected so that the actual question of interest in an experiment may be pursued (e.g., Cornero et al., 2020; Tebbich et al., 2007). Here, some birds were prone to side preferences (Plato, Fry, and Connelly), whereas others were not (Leo, Huxley). In this study, side preferences were only corrected intentionally by the experimenter (by forcing a choice to the other side) when noted in the text (see Results, Fig. 9), otherwise minor preferences self-resolved, or were severe enough to be unbreakable (Plato in Task 7). It is not known what may make some individuals more prone to side preferences, or even if there truly are individual differences in this propensity, although anecdotally it does seem so. Any individual differences in the likelihood of side preferences may have masked more complex OP in some individuals. On the other hand, the side preferences may be a result rather than a cause, and may indicate a strategy by a bird unable or unwilling to solve the task as designed, for reasons such as neophobia, rule-learning from past experiences, noncompliance, lack of interest, or increasing cognitive load (see Cornero et al., 2020).

Connelly, specifically, has been reported to make odd choices and be prone to side preferences (Tebbich et al., 2007): He even made incorrect choices over a sustained period during a trap-tube experiment, to a statistically significant degree. Tebbich et al. (2007) concluded he must have abstracted some rule about the task, but which rule it was or why was not determined. In the present study he was also prone to side preferences, and although he performed at chance rather than significantly incorrectly, it is possible he may have been using some undetectable cue or an unknown rule to make his decisions on the tasks he failed.

### Past experiences

It is likely to be evolutionarily advantageous for individual animals to learn from their environment and experiences: When animals can act on their environments, they must be able to encode any regularities, and may learn cause and effect by being exposed to these, leading to “folk physics” and problem solving abilities including tool use (i.e., Tebbich et al., 2007). Likewise, what individuals have or have not experienced when raised in experimental settings may affect OP performance (Pepperberg & Funk, 1990; Pepperberg & Kozak, 1986). Some individual rooks tested in this study have had experience with formal physical, tool use, and problem-solving tasks (at least Fry and Connelly, but likely more; Bird & Emery, 2009a, 2009b; Buitendijk et al., 2010; Seed et al., 2006; Tebbich et al., 2007). In one pair of studies involving original and modified trap-tube tasks, it was hypothesized that past experience with the original tube may have affected the performance of some on a modified set of trap-tubes, because they performed worse than another set of rooks that received the modified trap-tubes without experiencing the original tube: They may have learned a rule during exposure which was not beneficial to solving the transfer task, and could not let it go (Connelly was one of the birds offered the original tube and then the transfer, whereas Fry received the transfer task first; Seed et al., 2006; Tebbich et al., 2007). Leo, a younger rook who was not alive then, has not received extended experience with tool-use, problem solving, or physical tasks, and may not have been bound by such experiences.

Finally, it is important to note that this sample of rooks had exhibited extremely strong neophobic responses to new experimenters and setups in their adult age, which required a long habituation period before this study was possible, and made a few allowances necessary (see Methods and Supplementary Material). All the factors involved are not known, although the one-time collapse of a tree onto their aviary and its destruction and rebuilding is likely to have played a large role (N.S.C., personal observation). Regardless, testing this sample, especially on this first experiment with F.M.C., was challenging. Yet most of the surviving rooks have remained testable since, and others have begun to participate in later experiments (Aristotle, Cassandra). Therefore, we argue there is value in persisting with a modified study, made to be as stringent as the subjects allow, for the collection of data and advancement towards future experiments that would otherwise have remained out of reach.

## Conclusions

In conclusion, rooks show object permanence abilities as complex as those of other bird species studied to date, including other species of corvids and parrots. Taken together, even with the most conservative interpretation, these rooks could pass the first five stages of OP, and Leo completed all six, demonstrating the potential for rooks to understand multiple invisible displacements. Like some other corvids, it is likely that rooks may benefit from these abilities to successfully pilfer other birds’ food caches, as well as to protect their own caches from pilferage. They would also require strong OP abilities to keep track of their depleted, pilfered, or recached caches in the winter, a strong evolutionary advantage for surviving long periods of inclement weather in their environment. Additional investigation into the OP abilities of caching and noncaching bird species could pinpoint whether OP abilities are derived separately in corvids, for the purposes of caching, and in parrots, for some other purpose, or if they may be an ancestral ability present in many more avian species.

## Supplementary Information

Below is the link to the electronic supplementary material.

Supplementary file1 (PDF 10.6 MB)

## Data Availability

All data generated or analyzed during this study are included in this published article and its supplementary files. Additionally, raw data and videos are available from the corresponding author on reasonable request.

## Footnotes

1 Note that Plato was the only bird to receive Task 6 before 5, as initially Tasks 5 and 11 were to be omitted, and “A-not-B” errors not to be examined, given their confusing relevance. Given Plato’s performance on Task 6, it was decided to give him Task 5 after his Task 6, and to give the other birds Task 5 in the normal order.

## Code availability

No code was used to analyze this data.

## References

[CR1] Auersperg, A. M., Szabo, B., von Bayern, A. M., & Bugnyar, T. (2014). Object permanence in the Goffin cockatoo (*Cacatua goffini*). *Journal of Comparative Psychology,**128*(1), 88–98. 23875920 10.1037/a0033272

[CR2] Bird, C. D., & Emery, N. J. (2008). Using video playback to investigate the social preferences of rooks. *Corvus frugilegus. Animal Behaviour,**76*(3), 679–687.

[CR3] Bird, C. D., & Emery, N. J. (2009a). Insightful problem solving and creative tool modification by captive nontool-using rooks. *Proceedings of the National Academy of Sciences,**106*(25), 10370–10375. 10.1073/pnas.0901008106 PMC2700937 19478068

[CR4] Bird, C. D., & Emery, N. J. (2009b). Rooks use stones to raise the water level to reach a floating worm. *Current Biology,**19*(16), 1410–1414. 19664926 10.1016/j.cub.2009.07.033

[CR5] Bird, C. D., & Emery, N. J. (2010). Rooks perceive support relations similar to six-month-old babies. *Proceedings of the Royal Society B: Biological Sciences,**277*(1678), 147–151. 10.1098/rspb.2009.1456 PMC2842627 19812083

[CR6] Bremmer, J. G. (1978a). Egocentric versus allocentric spatial coding in nine-month-old infants: Factors influencing choice of code. *Developmental Psychology,**14*, 346–355.

[CR7] Bremmer, J. G. (1978b). Spatial errors made by infants: Inadequate spatial cues or evidence of egocentrism. *British Journal of Psychology,**69*, 77–84. 626806 10.1111/j.2044-8295.1978.tb01634.x

[CR8] Brodbeck, D. R. (1994). Memory for spatial and local cues: A comparison of a storing and a nonstoring species. *Animal Learning & Behavior,**22*(2), 119–133.

[CR9] Bugnyar, T., Stoewe, M., & Heinrich, B. (2007). The ontogeny of caching in ravens, Corvus corax. *Animal Behaviour,**74*(4), 757–767.

[CR10] Buitendijk, C., Cheke, L. G., Emery, N. J., & Clayton, N. S. (2010) *Folk physics in rooks: A test on shape matching*. Unpublished thesis in Clayton’s lab.

[CR11] Butterworth, G., Jarrett, N., & Hicks, L. (1982). Spatiotemporal identity in infancy: Perceptual competence or conceptual deficit? *Developmental Psychology, IS,**18*, 435–449.

[CR12] Call, J. (2001). Object permanence in orangutans (*Pongo pygmaeus*), chimpanzees (*Pan troglodytes*), and children (*Homo sapiens*). *Journal of Comparative Psychology,**115*(2), 159–171. 11459163 10.1037/0735-7036.115.2.159

[CR13] Chevalier-Skolnikoff, S. (1976). The ontogeny of primate intelligence and its implications for communicative potential: A preliminary report. In S. R. Hamad, H. D. Steklis, & J. Lancaster (Eds.), *Annals of the New York Academy of Sciences: Origins and evolution of language and speech* (vol. 280, pp. 173–211). New York Academy of Sciences.

[CR14] Clayton, N. S. (1992). The ontogeny of food-storing and retrieval in marsh tits. *Behaviour,**122*(1/2), 11–25.

[CR15] Clayton, N. S. (1994). The role of age and experience in the behavioural development of food-storing and retrieval in marsh tits. *Parus palustris. Animal Behaviour,**47*(6), 1435–1444. 10.1016/0166-4328(95)00049-6 8851924

[CR16] Clayton, N. S., & Krebs, J. R. (1993). Lateralization in *Paridae*: Comparison of a storing and a non-storing species on a one-trial associative memory task. *Journal of Comparative Physiology A,**171*(6), 807–815.

[CR17] Clayton, N. S., & Krebs, J. R. (1994a). Memory for spatial and object-specific cues in food-storing and non-storing birds. *Journal of Comparative Physiology A,**174*(3), 371–379.

[CR18] Clayton, N. S., & Krebs, J. R. (1994b). Hippocampal growth and attrition in birds affected by experience. *Proceedings of the National Academy of Sciences,**91*(16), 7410–7414. 10.1073/pnas.91.16.7410 PMC44410 8052598

[CR19] Cornero, F. M., Hartsfield, L. A., & Pepperberg, I. M. (2020). Piagetian liquid overconservation in grey parrots (*Psittacus erithacus*). *Journal of Comparative Psychology,**134*(2), 197–210. 31855033 10.1037/com0000209

[CR20] Dally, J. M., Emery, N. J., & Clayton, N. S. (2005a). Cache protection strategies by Western scrub-jays, *Aphelocoma californica*: Implications for social cognition. *Animal Behaviour,**70*(6), 1251–1263.

[CR21] Dally, J. M., Emery, N. J., & Clayton, N. S. (2005b). The social suppression of caching in Western scrub-jays (*Aphelocoma californica*). *Behaviour,**142*(7), 961–977.

[CR22] Dally, J. M., Clayton, N. S., & Emery, N. J. (2006a). The behaviour and evolution of cache protection and pilferage. *Animal Behaviour,**72*(1), 13–23.

[CR23] Dally, J. M., Emery, N. J., & Clayton, N. S. (2006b). Food-caching western scrub-jays keep track of who was watching when. *Science,**312*(5780), 1662–1665. 16709747 10.1126/science.1126539

[CR24] Dally, J., Clayton, N. S., & Emery, N. J. (2008). Social influences on foraging by rooks (*Corvus frugilegus*). *Behaviour,**145*(8), 1101–1124.

[CR25] Dally, J. M., Emery, N. J., & Clayton, N. S. (2010). Avian theory of mind and counter espionage by food-caching western scrub-jays (*Aphelocoma californica*). *European Journal of Developmental Psychology,**7*(1), 17–37.

[CR26] de Kort, S. R., Tebbich, S., Dally, J. M., Emery, N. J., & Clayton, N. S. (2006). The comparative cognition of caching. In E. A. Wasserman & T. S. Zentall (Eds.), *Comparative cognition: Experimental explorations of animal intelligence* (pp. 602–618). Oxford University Press.

[CR27] Doré, F. Y., Fiset, S., Goulet, S., Dumas, M.-C., & Gagnon, S. (1996). Search behavior in cats and dogs: Interspecific differences in working memory and spatial cognition. *Animal Learning & Behavior,**24*, 142–149.

[CR28] Emery, N. J., & Clayton, N. S. (2001). Effects of experience and social context on prospective caching strategies by scrub jays. *Nature,**414*(6862), 443–446. 11719804 10.1038/35106560

[CR29] Emery, N. J., Seed, A. M., von Bayern, A. M., & Clayton, N. S. (2007). Cognitive adaptations of social bonding in birds. *Philosophical Transactions of the Royal Society B: Biological Sciences,**362*(1480), 489–505. 10.1098/rstb.2006.1991 PMC2346513 17255008

[CR30] Funk, M. S. (1996). Development of object permanence in the New Zealand parakeet (*Cyanoramphus auriceps*). *Animal Learning & Behavior,**24*, 375–383.

[CR31] Gagnon, S., & Doré, F. Y. (1994). Cross-sectional study of object permanence in domestic puppies (*Canis familiaris*). *Journal of Comparative Psychology,**108*(3), 220–232. 7924252 10.1037/0735-7036.108.3.220

[CR32] Garamszegi, L. Z., & Eens, M. (2004). The evolution of hippocampus volume and brain size in relation to food hoarding in birds. *Ecology Letters,**7*(12), 1216–1224.

[CR33] Goldfield, E. C., & Dickerson, D. J. (1981). Keeping track of locations during movement in 8- to 10-month old infants. *Journal of Experimental Child Psychology,**32*, 48–64.

[CR34] Gómez, J. C. (2005). Species comparative studies and cognitive development. *Trends in Cognitive Sciences,**9*(3), 118–125. 15737820 10.1016/j.tics.2005.01.004

[CR35] Hampton, R. R., & Shettleworth, S. J. (1996). Hippocampus and memory in a food-storing and in a nonstoring bird species. *Behavioral Neuroscience,**110*(5), 946–964. 8918998 10.1037//0735-7044.110.5.946

[CR36] Healy, S. D., Clayton, N. S., & Krebs, J. R. (1994). Development of hippocampal specialisation in two species of tit (Parus spp.). *Behavioural Brain Research,**61*(1), 23–28. 8031493 10.1016/0166-4328(94)90004-3

[CR37] Healy, S. D., & Krebs, J. R. (1992a). Comparing spatial memory in two species of tit: Recalling a single positive location. *Animal Learning & Behavior,**20*(2), 121–126.

[CR38] Healy, S. D., & Krebs, J. R. (1992b). Food storing and the hippocampus in corvids: Amount and volume are correlated. *Proceedings of the Royal Society of London Series B: Biological Sciences,**248*(1323), 241–245.

[CR39] Healy, S. D., & Krebs, J. R. (1993). Development of hippocampal specialisation in a food-storing bird. *Behavioural Brain Research,**53*(1/2), 127–131. 8466658 10.1016/s0166-4328(05)80272-4

[CR40] Healy, S. D., & Krebs, J. R. (1996). Food storing and the hippocampus in *Paridae*. *Brain, Behavior and Evolution,**47*(4), 195–199. 9156782 10.1159/000113239

[CR41] Hoffmann, A., Rüttler, V., & Nieder, A. (2011). Ontogeny of object permanence and object tracking in the carrion crow. *Corvus corone. Animal Behaviour,**82*(2), 359–367.

[CR42] Jaakkola, K. (2014). Do animals understand invisible displacement? A critical review. *Journal of Comparative Psychology,**128*(3), 225–239. 24611640 10.1037/a0035675

[CR43] Jolles, J., Ostojić, L., & Clayton, N. S. (2013). Dominance, pair bonds and boldness determine social foraging tactics in rooks (*Corvus frugilegus*). *Animal Behaviour,**85*(6), 1261–1269.

[CR44] Källander, H. (2007). Food hoarding and use of stored food by rooks. *Bird Study,**54*(2), 192–198.

[CR45] Kamil, A. C., & Balda, R. P. (1985). Cache recovery and spatial memory in Clark’s nutcrackers (*Nucifraga columbiana*). *Journal of Experimental Psychology: Animal Behavior Processes,**11*(1), 95–111.

[CR46] Kamil, A. C., Balda, R. P., & Olson, D. J. (1994). Performance of four seed-caching corvid species in the radial-arm maze analog. *Journal of Comparative Psychology,**108*(4), 385–393. 7813195 10.1037/0735-7036.108.4.385

[CR47] Kamil, A. C., Balda, R. P., & Good, S. (1999). Patterns of movement and orientation during caching and recovery by Clark’s nutcrackers. *Nucifraga columbiana. Animal Behaviour,**57*(6), 1327–1335. 10373267 10.1006/anbe.1999.1112

[CR48] Krebs, J. R. (1990). Food-storing birds: Adaptive specialization in brain and behaviour? *Philosophical Transactions of the Royal Society of London Series B: Biological Sciences,**329*(1253), 153–160. 1978360 10.1098/rstb.1990.0160

[CR49] Klerk, S., & Jacobs, I. (2021). Reasoning by exclusion. In J. Vonk & T. K. Shackelford (Eds.), *Encyclopedia of animal cognition and behavior. *Springer Nature.

[CR50] Krebs, J. R., Clayton, N. S., Healy, S. D., Cristol, D. A., Patel, S. N., & Jolliffe, A. R. (1996). The ecology of the avian brain: Food-storing memory and the hippocampus. *Ibis,**138*(1), 34–46.

[CR51] Lucas, J. R., Brodin, A., de Kort, S. R., & Clayton, N. S. (2004). Does hippocampal size correlate with the degree of caching specialization? *Proceedings of the Royal Society of London Series B: Biological Sciences,**271*(1556), 2423–2429. 10.1098/rspb.2004.2912 PMC1523289 15590591

[CR52] Mathieu, M., Bouchard, M. A., Granger, L., & Herscovitch, J. (1976). Piagetian object-permanence in *Cebus capucinus*, *Lagothrica flavicauda* and *Pan troglodytes*. *Animal Behaviour,**24*(3), 585–588.

[CR53] Okamoto-Barth, S., & Call, J. (2008). Tracking and inferring spatial rotation by children and great apes. *Developmental Psychology,**44*(5), 1396–1408. 18793071 10.1037/a0012594

[CR54] Olson, D. J., Kamil, A. C., Balda, R. P., & Nims, P. J. (1995). Performance of four-seed caching corvid species in operant tests of nonspatial and spatial memory. *Journal of Comparative Psychology,**109*(2), 173–181. 7758292 10.1037/0735-7036.109.2.173

[CR55] Osthaus, B. (2021). A-not-B problem. In J. Vonk & T. K. Shackelford (Eds.), *Encyclopedia of animal cognition and behavior. *Springer Nature.

[CR56] Pepperberg, I. M., & Kozak, F. A. (1986). Object permanence in the African grey parrot (*Psittacus erithacus*). *Animal Learning & Behavior,**14*(3), 322–330.

[CR57] Pepperberg, I. M., & Funk, M. S. (1990). Object permanence in four species of psittacine birds: An African grey parrot (*Psittacus erithacus*), an Illiger mini macaw (*Ara**maracana*), a parakeet (*Melopsittacus undulatus*), and a cockatiel (*Nymphicus hollandicus*). *Animal Learning & Behavior,**18*(1), 97–108.

[CR58] Pepperberg, I. M., Willner, M. R., & Gravitz, L. B. (1997). Development of Piagetian object permanence in grey parrot (*Psittacus erithacus*). *Journal of Comparative Psychology,**111*(1), 63–75. 9090138 10.1037/0735-7036.111.1.63

[CR59] Pepperberg, I. M., Koepke, A., Livingston, P., Girard, M., & Hartsfield, L. A. (2013). Reasoning by inference: Further studies on exclusion in grey parrots (*Psittacus erithacus*). *Journal of Comparative Psychology,**127*(3), 272–281. 23421751 10.1037/a0031641

[CR60] Pepperberg, I. M., Gray, S. L., Mody, S., Cornero, F. M., & Carey, S. (2019). Logical reasoning by a grey parrot? A case study of the disjunctive syllogism. *Behaviour,**156*(5/8), 409–445.

[CR61] Piaget, J. (1952). *The origins of intelligence in children (M. Cook, Trans.).* International Universities Press.

[CR62] Piaget, J. (1954). *The construction of reality in the child (M. Cook, Trans.)*. Basic Books.

[CR63] Pollok, B., Prior, H., & Güntürkün, O. (2000). Development of object permanence in food-storing magpies (*Pica pica*). *Journal of Comparative Psychology,**114*(2), 148–157. 10890586 10.1037/0735-7036.114.2.148

[CR64] Prasad, A., Wood, S. M., & Wood, J. N. (2019). Using automated controlled rearing to explore the origins of object permanence. *Developmental Science,**22*(3), e12796. 30589167 10.1111/desc.12796

[CR65] Regolin, L., Vallortigara, G., & Zanforlin, M. (1994). Perceptual and motivational aspects of detour behaviour in young chicks. *Animal Behaviour,**47*(1), 123–131.

[CR66] Roberts, R. C. (1979). The evolution of avian food-storing behavior. *The American Naturalist,**114*(3), 418–438.

[CR67] Salwiczek, L. H., Emery, N. J., Schlinger, B., & Clayton, N. S. (2009). The development of caching and object permanence in Western scrub-jays (*Aphelocoma californica*): Which emerges first? *Journal of Comparative Psychology,**123*(3), 295–303. 19685971 10.1037/a0016303 PMC2836840

[CR68] Seed, A. M., Tebbich, S., Emery, N. J., & Clayton, N. S. (2006). Investigating physical cognition in rooks. *Corvus frugilegus. Current Biology,**16*(7), 697–701. 16581516 10.1016/j.cub.2006.02.066

[CR69] Seed, A. M., Clayton, N. S., & Emery, N. J. (2007). Postconflict third-party affiliation in rooks. *Corvus frugilegus. Current Biology,**17*(2), 152–158. 17240341 10.1016/j.cub.2006.11.025

[CR70] Seed, A. M., Clayton, N. S., & Emery, N. J. (2008). Cooperative problem solving in rooks (*Corvus frugilegus*). *Proceedings of the Royal Society B: Biological Sciences,**275*(1641), 1421–1429. 10.1098/rspb.2008.0111 PMC2602707 18364318

[CR71] Shaw, R. C., & Clayton, N. S. (2012). Eurasian jays, *Garrulus glandarius*, flexibly switch caching and pilfering tactics in response to social context. *Animal Behaviour,**84*(5), 1191–1200.

[CR72] Sherry, D. F., & Vaccarino, A. L. (1989). Hippocampus and memory for food caches in black-capped chickadees. *Behavioral Neuroscience,**103*(2), 308–318.

[CR73] Sophian, C. (1985). Understanding the movement of objects: Early developments in spatial cognition. *British Journal of Developmental Psychology,**3*, 321–333.

[CR74] Suddendorf, T., & Whiten, A. (2001). Mental evolution and development: Evidence for secondary representation in children, great apes, and other animals. *Psychological Bulletin,**127*(5), 629–650. 11548971 10.1037/0033-2909.127.5.629

[CR75] Tebbich, S., Seed, A. M., Emery, N. J., & Clayton, N. S. (2007). Non-tool-using rooks, *Corvus frugilegus*, solve the trap-tube problem. *Animal Cognition,**10*(2), 225–231. 17171360 10.1007/s10071-006-0061-4

[CR76] Triana, E., & Pasnak, R. (1981). Object permanence in cats and dogs. *Animal Learning & Behavior,**9*(1), 135–139.

[CR77] Ujfalussy, D. J., Miklósi, Á., & Bugnyar, T. (2013). Ontogeny of object permanence in a nonstoring corvid species, the jackdaw (Corvus monedula). *Animal Cognition,**16*(3), 405–416. 23161215 10.1007/s10071-012-0581-z PMC4417713

[CR78] Uzgiris, I. C., & Hunt, J. (1975). *Assessment in infancy: Ordinal scales of psychological development*. University of Illinois Press.

[CR79] Vander Wall, S. B. (1990). *Food hoarding in animals*. University of Chicago Press.

[CR80] Wang, L., Luo, Y., Maierdiyali, A., Chang, H., Ullah, S., & Li, Z. (2021). Azure-winged Magpies *Cyanopica cyanus* passed the tasks on the Uzgiris-Hunt scale of object permanence. *Journal of Ornithology,**162*(2), 605–613.

[CR81] Webster, M. M., & Rutz, C. (2020). How STRANGE are your study animals? *Nature,**582*(7812), 337–340. 32541916 10.1038/d41586-020-01751-5

[CR82] Zentall, T. R., & Raley, O. L. (2019). Object permanence in the pigeon (*Columba**livia*): Insertion of a delay prior to choice facilitates visible-and invisible-displacement accuracy. *Journal of Comparative Psychology,**133*(1), 132–139. 30382709 10.1037/com0000134

[CR83] Zewald, J., & Jacobs, I. (2021). Object permanence. In J. Vonk & T. K. Shackelford (Eds.), *Encyclopedia of animal cognition and behavior. *Springer Nature.

[CR84] Zucca, P., Milos, N., & Vallortigara, G. (2007). Piagetian object permanence and its development in Eurasian jays (*Garrulus glandarius*). *Animal Cognition,**10*(2), 243–258. 17242935 10.1007/s10071-006-0063-2

