# Supplementary material for: Object permanence in rooks (Corvus frugilegus): Individual differences and behavioral considerations
Source: Learn Behav. 2024 Sep 3;53(1):93–113. doi: 10.3758/s13420-024-00637-0 (PMC11880163; doi:10.3758/s13420-024-00637-0)
Supplement: Supplementary file 1 — Supplementary file1 (PDF 10.6 MB) [file 13420_2024_637_MOESM1_ESM.pdf]

Supplementary Materials

Avian OP Studies:

A list of the OP studies with bird species: *carrion crows*, Hoffmann, Rüttler & Nieder, 2011; *grey parrots*, Pepperberg & Funk, 1990; Pepperberg & Kozak; 1986; Pepperberg, Willner & Gravitz, 1997; *an Illiger mini macaw, a parakeet, and a cockatiel*, Pepperberg & Funk, 1990; *chickens,* Regolin, Vallortigara & Zanforlin, 1994; *chicks,* Prasad, Wood & Wood, 2019; *pigeons*, Zentall & Raley, 2019; *magpies*, Pollok, Prior & Güntürkün, 2000; *jackdaws*, Ujfalussy, Miklósi & Bugnyar, 2013; *Eurasian jays*, Zucca, Milos & Vallortigara, 2007; *Western scrub-jays*, Salwiczek et al., 2009; *Goffin's cockatoos*, Auersperg et al., 2014; *azure-winged magpies*, Wang et al., 2021; *ravens,* Bugnyar, Stoewe & Heinrich, 2007).

Subjects' Previous Testing Experiences:

Some of the subjects tested in this study had participated in previous experiments involving physical and social cognition: Bird & Emery, 2008; 2009a,b; 2010; Buitendijk et al., unpublished thesis in Clayton's lab examining shape-matching; Dally, Clayton & Emery, 2008; Emery et al., 2007; Jolles, Ostójic & Clayton, 2013; Seed, Clayton & Emery, 2007; 2008; Seed et al., 2006; Spierings et al., unpublished thesis in Clayton's lab examining female partners' motivational states; Tebbich et al., 2007.

Habituation Information:

This particular sample of birds had become highly neophobic towards new people and experimental materials in adulthood, and OP testing was additionally selected as their first

experiment after extended habituation to the main experimenter and the testing compartments (see *Discussion*) in order to both prepare them for and examine their readiness toward future experiments requiring similar materials and set-up. Should they be willing to participate in the OP tasks offered, they would be likely to participate in future, more complex experiments as well. However, rotation tasks often applied in conjunction with OP tasks (see Zewald & Jacobs, 2021) were omitted due to the logistical difficulty of presenting them to these highly neophobic birds.

Therefore, during about 100 days of habituation to the main experimenter (FMC) and testing space prior to this experiment, she noted when birds engaged in several habituation milestones, including increasing habituation to the experimenter, the testing space, and the testing materials, as well as the start of the current experiment and progression through the tasks (see main text, *Figure 10*). Post-hoc non-parametric Kruskal-Wallis tests on this indirectly recorded data, examining the effect of bird ID on either date of attainment or date of completion of the milestones examined were not significant ($p=0.9317$ and $p=0.9804$, respectively).

During the habituation period, FMC observed an interaction in which Leo displaced Plato from her, which she noted as significant because she had not observed this to have occurred unless Leo was displacing Plato away from Leo's mate, Cassandra. FMC then noted, subsequently, that Leo began to challenge Plato, although he was unlikely to attempt to displace him from the testing compartment as he did all other birds. By the end of the study, Plato had died and Leo was the obvious most dominant, a position he holds to date of writing; in about 400 days of unrelated testing for subsequent studies Leo was never observed to be flushed from the compartment by any other bird, although breeding season fights between him and Connelly are common. Instead, Leo has been observed to frequently and successfully flush every other bird out of the testing compartment.

OP - Detailed Procedures:

For all tasks, with the exception of the Olfactory Control, Tasks 5, 11, 16 and S(1) and S(2), a rook was considered to "pass" a task after recovering the hidden object 10 times, with no more than two errors (10/12 correct, p = 0.0386, two-tailed binomial test, chance at 0.5[1]). Alternatively, a rook could choose correctly on the first 6 trials in a row in these tasks and also be considered to be "at ceiling" and to have passed the task, to avoid boredom responses of overtraining. See Olfactory Control, Tasks 5, 11, 16, and S below for their specific pass criteria.

OLFACTORY CONTROL:

To ensure that rooks were unable to locate worms by smell, "blind" trials were conducted in which the rooks were shown that a worm was being hidden under one of two cups, but were not shown its hiding place. This task used two plastic cups (one marked with a red dot and one with a blue dot) upside down in a line, about 15 cm apart. The experimenter obscured the rooks' line of sight with a large, flat piece of cardboard, then showed that a waxworm was being hidden behind this blind, but without indicating where: to accomplish this, the experimenter directed the rooks' attention to the worm over the blind, then lowered it behind the blind, and sequentially lifted and replaced each cup, depositing the worm under one surreptitiously (side of cup colours and location of hiding were pseudo-randomized for each individual, with the condition that a worm not be hidden under the same cup more than twice in a row). After the worm was hidden, the experimenter

---

[1] Chance is conservatively left at 0.5 even for Tasks which include 3 possible hiding locations due to the difficulty of assigning probabilities to each action the bird might choose to take (as described in Pollok, Prior & Güntürkün, 2000). As such, the usual binomial chance of correct vs. incorrect is used here throughout.

lowered the blind, and allowed the rooks to choose one cup: rooks were allowed to eat any worm they happened to find.

Each rook could experience up to 12 trials in this manner, and it was concluded that they could not rely on olfactory cues to locate rewards if their performance was not significantly better than chance (50%, two-tailed binomial). Additionally, rooks that refused to choose a cup from the beginning, or became unwilling to participate during the course of the experiment (failing to open a cup within 5 minutes, either initially or after successfully opening at least one cup), were also considered to be unable to find the worm by olfaction, and further testing on this task was discontinued to avoid making them uncompliant for the rest of the experiment. Rooks did not all receive the olfactory control in the same order among their tasks, but all that received it had it before receiving Task 10.

TASK 4: Finding an object that is completely covered.

As in all tasks, rooks were shown a single waxworm when they entered the testing area (*Supplemental Figure 1*). The experimenter made sure the rook had seen the worm and demonstrated interest in it, for example by examining it with one eye or leaning towards it. The experimenter then covered the worm with a small plastic cup, placed upside down (*Supplemental Figure 2*). In order to respond correctly, rooks had to approach the cup, remove the cup either by lifting it or pecking it away, and take the worm. Errors were: showing interest in the cup but failing to remove it (such as approaching and pecking around it), or failing to approach the cup within five minutes without demonstrating the signs of fear or lack of motivation described above.

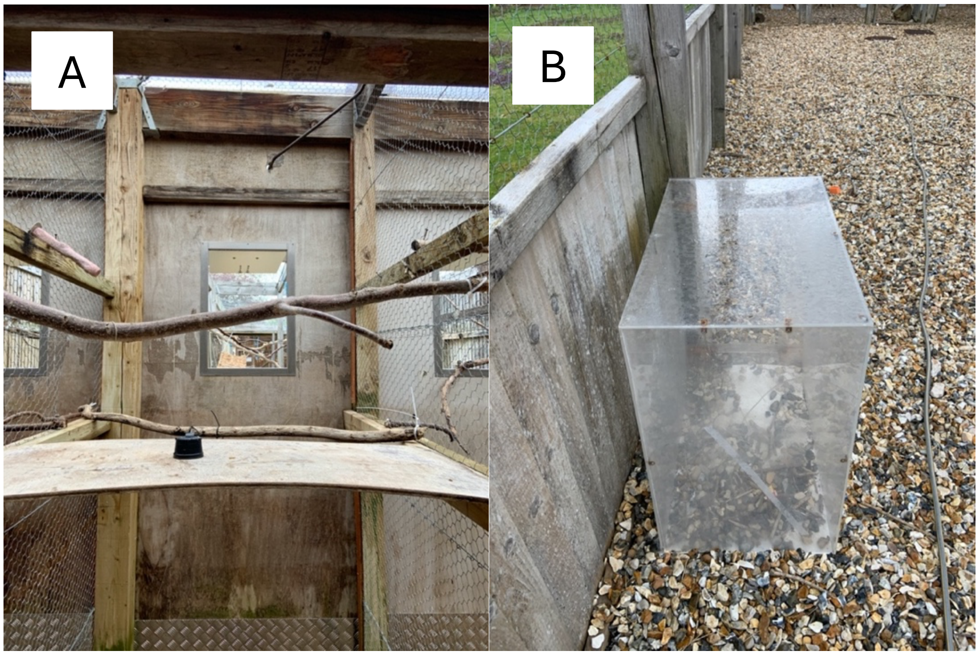

Supplemental Figure 1. *(A)* The testing compartment, surface, and sample cup (similar to Task 4), from experimenter's perspective; *(B)* The testing surface in the aviary, from experimenter's perspective.

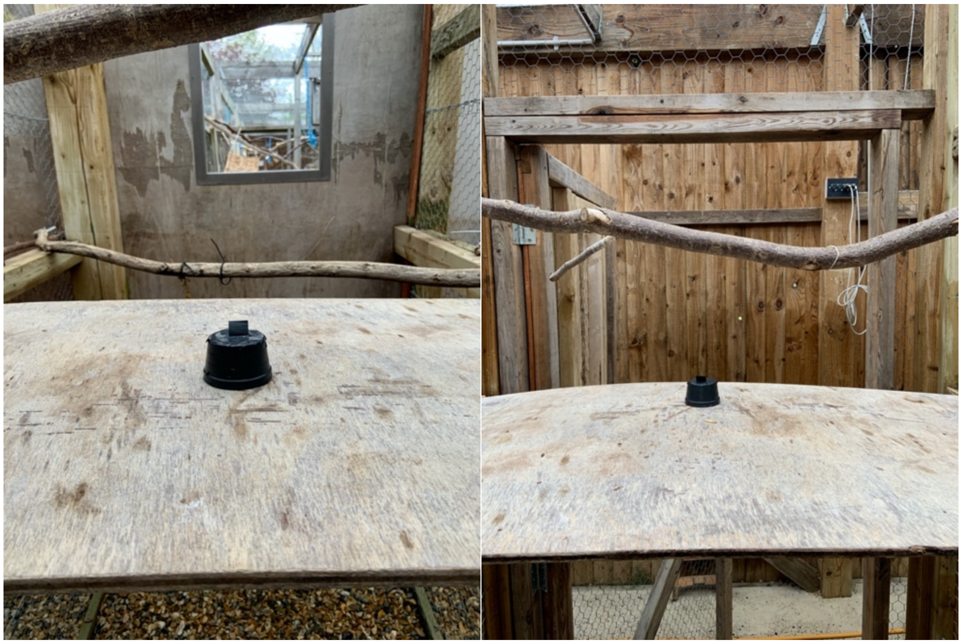

Supplemental Figure 2. The experimental setup for Task 4 inside the compartment; *Left:* from the experimenter's perspective; *Right:* from the bird's perspective (waxworm pictured).

TASK 5: Finding an object completely covered in two places.

Rooks were presented with two cups, as in the olfactory control. The experimenter then placed a waxworm on the table and covered it with one; the side of baiting (experimenter's left or right) was randomized for each individual (*Supplemental Figure 3*). If the rook chose correctly, the experimenter repeated the baiting procedure, hiding another worm under the same side. If the rook was correct again, the experimenter repeated the baiting procedure, but this time hid the worm under the other, previously un-baited cup on the other side. In order to be correct, rooks had to search in the new hiding place, rather than persevere. In the next round, the order of hiding was reversed, so that the reward was hidden twice under the new location, and then again in the first location the third time, and so on. Rooks were considered to pass the task if they completed six rounds without making "A-not-B" errors within five days of testing, or if they completed three rounds in a row without making any "A-not-B" errors. Any "A-not-B" errors witnessed were noted, and any not "A-not-B" errors (choosing the incorrect cup in the first or second presentation instead of the third) led to the trial set being considered a mistrial. Such a trial set was restarted, and this rarely occurred.

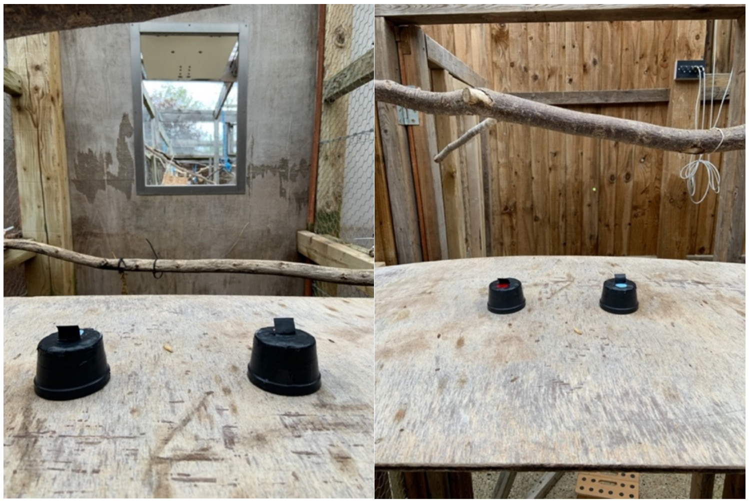

Supplemental Figure 3. The experimental setup for Tasks 5 and 6 inside the compartment; *Left:* from the experimenter's perspective; *Right:* from the bird's perspective (waxworm pictured in both).

TASK 6: Finding an object completely covered in two places alternately.

Rooks were presented with the same setup as in Task 5, but this time the location of hiding alternated: When the rook recovered a worm correctly, the experimenter repeated the baiting procedure, hiding another worm under the opposite-side cup. Once the rook recovered this worm again, the experimenter repeated the baiting procedure, but this time hid the worm under the first cup, and so on (*Supplemental Figure 3*). The initial side of baiting, as well as the side each colour cup was on, were randomized for each individual, such that the same colour-cup was not correct more than twice in a row. Errors were defined as in Task 4, with the addition of uncovering the incorrect cup.

TASK 7: Finding an object completely covered in three places.

Rooks were presented with three identical cups, of the same type as those used in Tasks 4-6, placed upside down in a line on the table and about 15 cm apart from each other. The additional cup was marked with a dot of yellow paint. The experimenter placed a waxworm under one of the cups, completely hidden – location of baiting (left, center or right) and colour of baited cup (red, blue, or yellow) were randomized for each individual rook, with the location of hiding never being repeated twice in a row (*Supplemental Figure 4*).

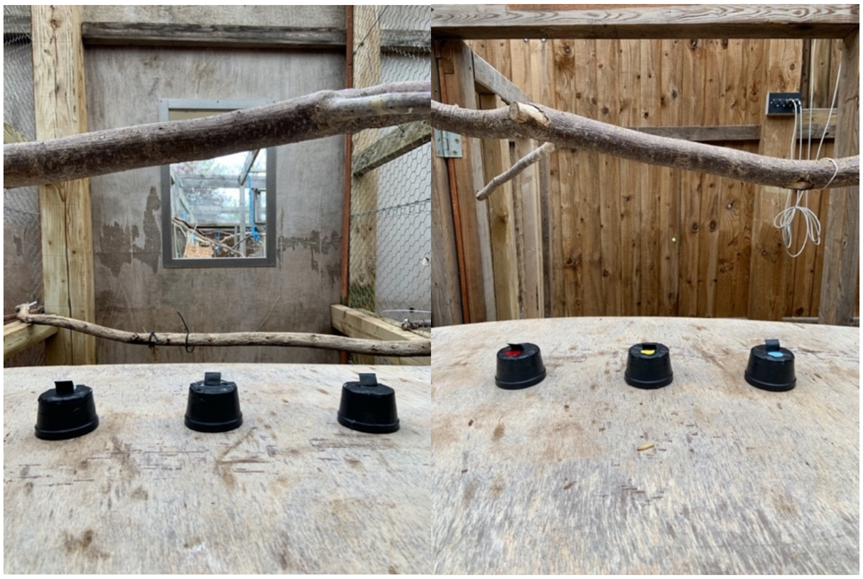

Supplemental Figure 4. The experimental setup for Tasks 7 and 8 inside the compartment; *Left:* from the experimenter's perspective; *Right:* from the bird's perspective (waxworm pictured).

TASK 8: Finding an object after successive visible displacements.

Rooks were presented with the same setup as in Task 7. The experimenter then placed a waxworm under one of the cups but then immediately lifted the cover and visibly transferred the worm to the second cup in the sequence, but then immediately lifted the cover and visibly transferred the worm to the third cup in the sequence – order of presentation (starting from left or right), and position of colours on the line, were randomized for each individual rook (*Supplemental Figure 4*). The order of transfer was always linear from left to right.

TASK 9: Finding an object under three superimposed screens.

In the interest of time, this task was skipped as it was considered redundant – it requires covering a worm with three cups of slightly increasing size, with the passing criterion being that a rook persevere in removing all three cups to obtain the worm under the last cup. Passing one task is indicative of capacity of solving earlier tasks (Pepperberg & Funk, 1990), and since this task is

conceptually similar to Task 4 but requires increased motivation and enduring attention, it would be justifiable to skip it. This would also prevent logistical issues associated with finding three cups of slightly increasing sizes that were just right so that a rook could lift each one over the ones below it and not be neophobic towards the additional number of cups or increasing size of the cups.

TASK 10: Finding an object following one invisible displacement.

Rooks were presented with a single waxworm. The experimenter then covered the worm with the unmarked cup used in Task 4, and then slid the cup (and worm) behind an upright cardboard screen, ~20x17 cm in size. The experimenter surreptitiously left the worm behind the screen, then continued sliding the (now empty) upside down cup off to the other side (the side of motion was pseudo-randomized for each trial with no more than two in a row having the same pattern; *Supplemental Figure 5*). The experimenter then lifted the cup, and showed the rook that it was empty.

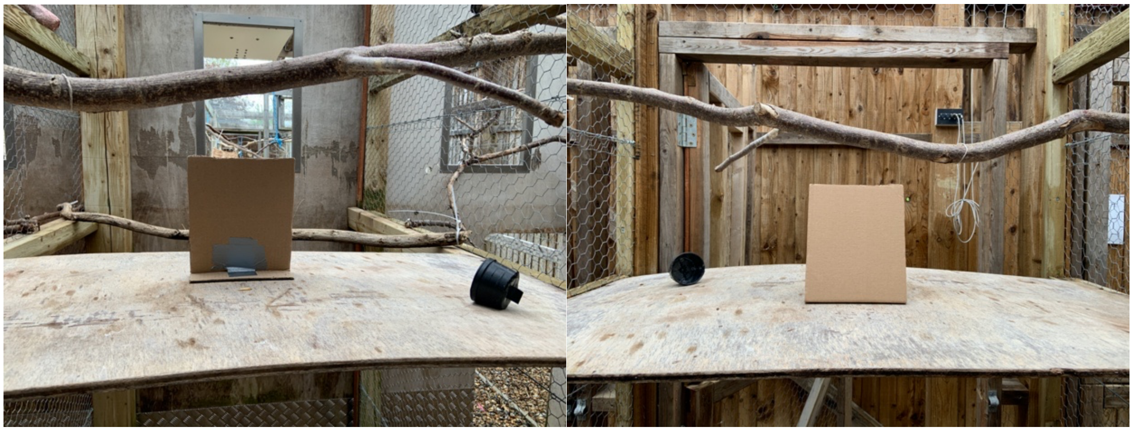

Supplemental Figure 5. The experimental setup for Task 10 inside the compartment; *Left:* from the experimenter's perspective (waxworm pictured); *Right:* from the bird's perspective.

TASK 11: Finding an object following one invisible displacement with two screens.

Rooks were shown a single waxworm, between two upright cardboard screens (of the same type as in Task 10), placed 15cm apart, with a large cardboard divider placed upright between them, so the rook could not walk between both screens to choose (resulting in possible confusion regarding their choice). The screens were marked with colourful dots in the same manner as the cups in the other tasks, but due to difficulty in quickly removing these upright screens without scaring off a participant bird, the side each colour screen was on was static for each bird (*Supplemental Figure 6*). The experimenter then covered the worm with an unmarked cup, and then slid the cup (and worm) behind one of the two cardboard screens. The experimenter surreptitiously left the worm behind this screen, then continued sliding the (now empty) upside down cup off to the other side of the screen. The experimenter then lifted the cup, and showed that it was empty. As in Task 5, the worm was hidden twice in one location (randomized for each bird), and then hidden in the other location on the third trial. In order to not demonstrate "A-not-B" errors, rooks had to search in the new hiding place, rather than persevere. In the next round, the order of hiding was reversed, so that it was hidden twice under the new location, and then again in the first location the third time, and so on. Rooks were considered to pass the task if they completed six rounds without making "A-not-B" errors within five days of testing, or by completing three rounds in a row without making "A-not-B" errors. Any "A-not-B" errors were noted. Any not "A-not-B errors" (erring in the first or second presentation to the same side, before the switch) were counted as mistrials and repeated (with the exception of Fry, due to the large number of occurrences of such errors, for which they are reported; see *Supplementary Materials – Individual Trial Outcomes).*

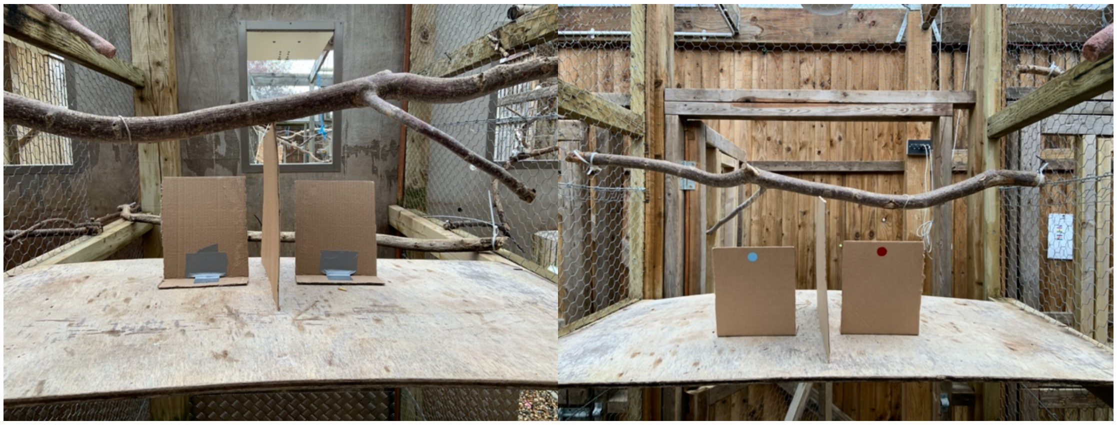

Supplemental Figure 6. The experimental setup for Tasks 11 and 12 inside the compartment; *Left:* from the experimenter's perspective (waxworm pictured); *Right:* from the bird's perspective.

TASK 12: Finding an object following one invisible displacement with two screens alternated.

Rooks were presented with the same setup as in Task 11. The experimenter then behaved as in Task 11, but as in Task 6, the worm was hidden in alternate locations (initial side randomized for each bird; *Supplemental Figure 6*).

TASK 13: Finding an object following one invisible displacement with three screens.

Rooks were presented with three upright cardboard screens (of the same type and colour markings as in the previous tasks), placed in a line on the table. Screens now had attached side flaps, 20cm tall and 5 cm wide, to prevent a rook seeing behind all screens at once when it moved forward to make a choice. The experimenter then covered the worm with an unmarked cup, and slid the cup (and worm) behind one of three screens (*Supplemental Figure 7*). The experimenter behaved as in Task 11, but as in Task 7, the worm was hidden in alternate locations (randomized for each bird).

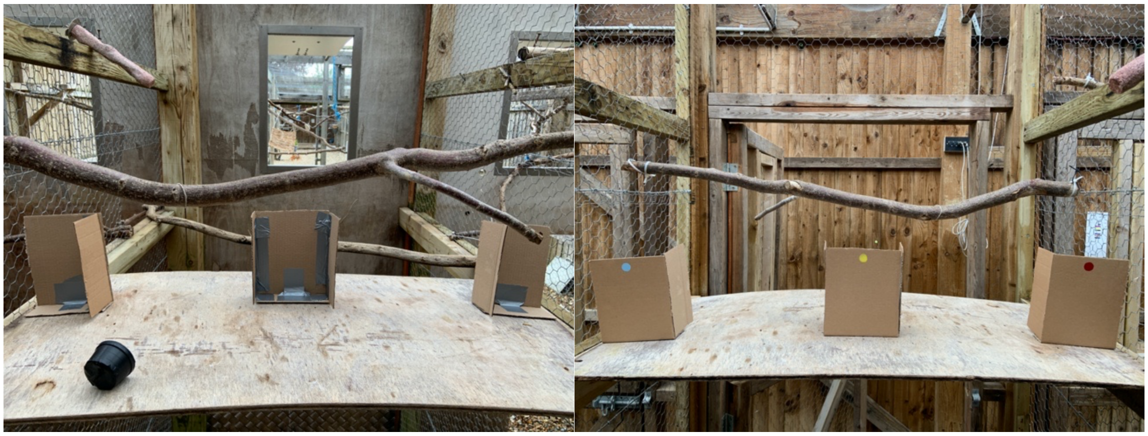

Supplemental Figure 7. The experimental setup for Tasks 13, 14, 15 and 16 inside the compartment; *Left:* from the experimenter's perspective (waxworm pictured); *Right:* from the bird's perspective.

<u>TASK 14: Finding an object following a series of invisible displacements.</u>

Rooks were presented with the same setup as in Task 13. The experimenter then placed a waxworm on one hand, closed the hand, and passed it behind the three screens. The direction of motion was randomized for each trial, such that no more than two in a row followed the same direction (*Supplemental Figure 7*). The direction of motion was always linear from left to right or right to left. The experimenter left the worm behind the last screen, and then showed the empty hand to the bird. Rooks were considered to pass the task if they correctly retrieved the worm by either checking the last screen first, or checking the screens in order of presentation. Randomly interspersed in these trials were control trials in which the experimenter left the worm behind either the first or second screens, and then immediately showed the empty hand to the bird, before continuing to pass behind the remaining screens. To indicate that it was not simply searching in the last place the experimenter touched, the bird should not have sought the worm behind the last screen in these cases.

TASK 15: Finding an object following a series of invisible displacements with evidence of representation.

Rooks were presented with the same setup as in Task 14 (*Supplemental Figure 7*). The experimenter then behaved as in Task 14, but left the worm behind the *first* screen, and then showed the empty hand to the bird only after it left the last screen. The direction of motion was always linear from left to right or right to left. Rooks were considered to pass the task if they went where they expected the worm (by checking the last screen first).

TASK 16: Finding an object following a series of invisible displacements with evidence of representation by substitution.

Rooks were presented with the same setup as in Task 15. The experimenter then behaved as in Task 15, but surreptitiously left either a mealworm or a peanut (which are not nearly as desirable as a waxworm) behind the first screen instead, and then showed the empty hand to the bird (*Supplemental Figure 7*). This task is intended to generate surprise or frustration at encountering an unexpected, less-desirable reward: it may be evidence of secondary representation of the object, rather than a simple knowledge that *something* is missing (Pepperberg, Willner & Gravitz; 1997). Rooks' behaviour was monitored for their reaction to finding a different, less-preferred food item than they were initially shown; rooks may refuse to consume the worm, throw the worm at the experimenter, show a startle reaction, examine the worm, surrounding area, and experimenter, and even leave the compartment without the reward.

<u>TASK S: Shell Game.</u>

Rooks were presented with a single waxworm. For S(1), The experimenter then covered the worm with one of three upside-down cups, as in Task 7, and then proceeded to slide this cup to swap positions with one of the remaining two cups, in a randomized order (*Supplemental Figure 4*). Rooks were considered to pass the task if they correctly retrieved the worm 12/15 times (errors are defined as in Task 7; p = 0.0352, two-tailed binomial test, chance at 0.5). Among these trials were control trials, randomly interspersed, in which the two un-baited cups swapped positions, and the baited cup remained still. Rooks should not have been fooled into choosing one of the cups moved to show they had not simply learned to choose one of the swapped cups. For S(2), Leo was also given a second version of Task S, in which two swaps occurred: either both involving the baited cup, only one involving the baited cup (either in the first swap, or in the second swap), or neither swap involving the baited cup. He received 24 trials in this manner, with criterion being 18/24 trials correct (p = 0.227, two-tailed binomial test, chance at 0.5).

Additional notes:

A handful of early sessions were conducted in the aviary because the beginning of testing coincided with breeding season, and some birds became unwilling to enter the compartments then (see main text, *Figure 9* for notes). However, other birds were always at least about 3m away from the testing surface when birds were tested in the aviary (*Supplemental Figure 1B*). Another exception is Huxley, who would be flushed out of the compartment by other birds if she came in for testing without her mate, Plato. Noting this, Plato was allowed to remain on the hatch while Huxley worked. Almost all of Plato's sessions on tasks he received occurred before Huxley's (with the exception of the latter half of Task 7, which he did not pass in any case), so he could not have

learned from her. Additionally, as many components of each trial as possible were randomized between individuals (see *Detailed OP Methods*), such that even birds observing another bird (when visual isolation could not fully occur) would have serious difficulty learning the tasks by observation.

Though the experimenter could not see a baited worm behind the vertical barriers from Leo's point of view, she became concerned that taller rooks (Connelly, Plato) might: Leo received Tasks 10 and 11 with smaller barriers, ~12x12cm as initially conceived, but after that they were enlarged as described (other birds, when applicable, were tested directly with the larger barriers). After habituating Leo to the larger barriers, two informal controls during Task 13, in which blinds were baited surreptitiously before he entered the compartment, were run to confirm the worm was invisible: he never went to the correct barrier first, and once never searched at all. Leo passed Task 12+ with the larger barriers directly. Fry would not habituate to Task 11 with the perpendicular barrier between the two options, so she was tested with the barriers with the side flaps.

Additionally, the experimenter noticed after running Tasks S(1) and S(2) with Leo that she had not thought to control for which hand passed over the other in every swap, so although Leo had passed both (S(1) in two rounds and S(2) in one), the experimenter later repeated these tasks by always passing the right hand over the left during any swap. For the second round of Leo's S(1) and for S(2), the data reported are for the newly-controlled repeat, showing no evidence that this had affected Leo's original performance (which, with the exception of the first round of S(1), was also passing: his original results were 14/15 for the second round of S(1) and 20/24 for S(2); because he passed his first repeated round of S(1) with 12/15, a second round was not given).

Finally, when a bird's behaviour was considered to be due to a side-preference (3 or more choices to the same side in a row, including at least 2 incorrect choices), the experimenter could

attempt to intentionally break this side preference by offering repeated trials to the opposite side until the bird finally switched to it, making a correct choice. If this procedure was attempted and was successful, the trials deemed to be due to side preferences were repeated from the beginning of the preference. Side-preferences encountered, any corrective action, and its outcome are described in the main text (see *Results*).

Supplementary Trial Videos:

https://osf.io/dq5mk/?view_only=3b5a84c60cfe402e9427cfe8660d49cf

Individual Trial Outcomes:

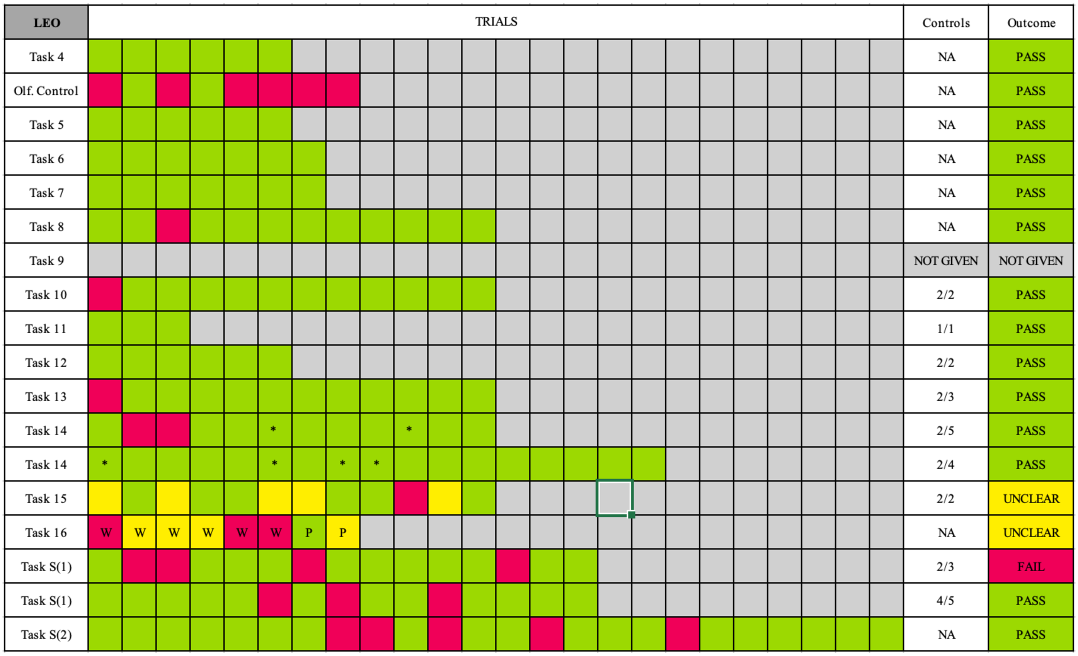

Supplementary Figure 8. Leo's results by trial given per task, with green indicating a correct response, yellow indicating an unclear or unexpected response, and red indicating an incorrect response. Asterisks in Task 14 indicate trials in which he went for the first blind first, as do yellow boxes in Task 15. "W" represents when he was given a mealworm, and "P" represents when the switch was done with a peanut. Also listed are control outcomes, with number correct out of the total, and the outcome of each task.

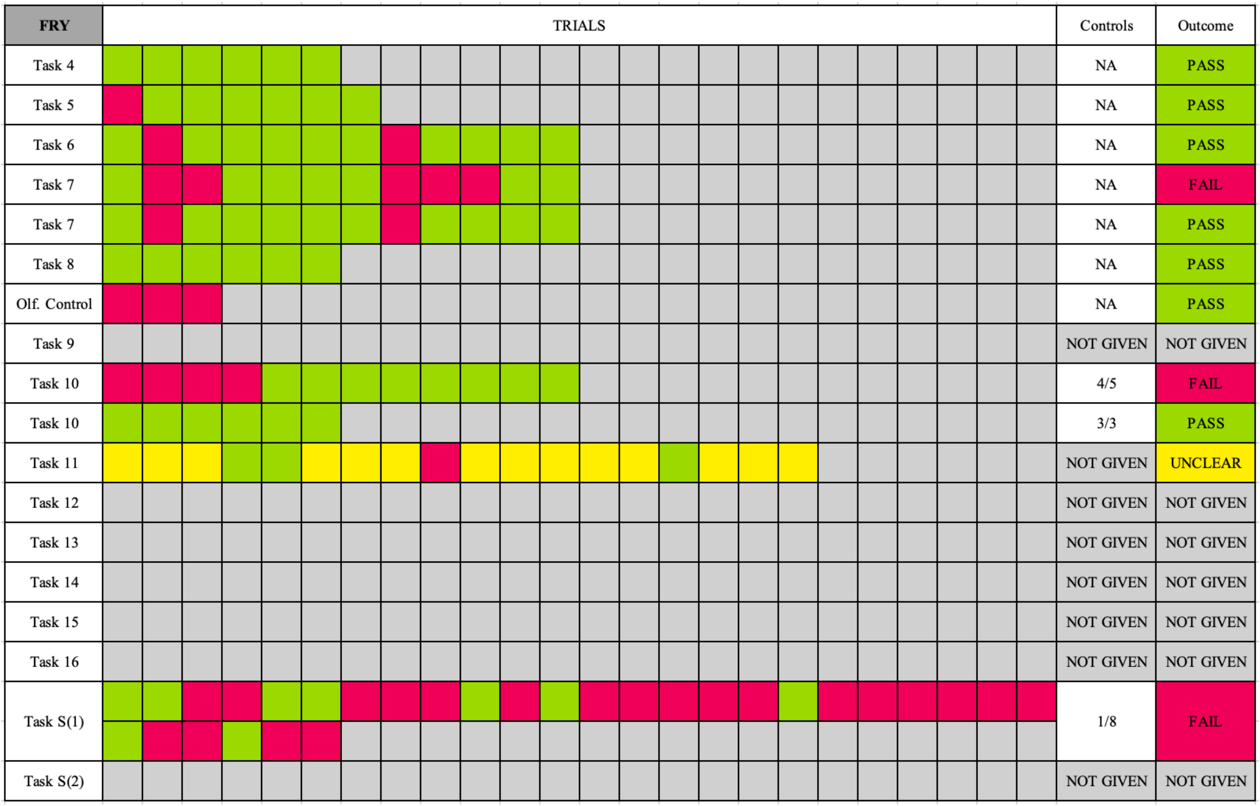

Supplementary Figure 9. Fry's results by trial given per task, with green indicating a correct response, yellow indicating an unclear or unexpected response, and red indicating an incorrect response. In Task 11, yellow trials are mistrials by making not "A-not-B" errors. Also listed are control outcomes, with number correct out of the total given, and the outcome of each task.

CONNELLY	TRIALS	Controls	Outcome
Task 4		NA	PASS
Olf. Control		NOT GIVEN	NOT GIVEN
Task 5		NA	FAIL
Task 5		NA	PASS
Task 6		NA	FAIL
Task 6		NA	FAIL
Task 6		NA	FAIL
Task 6		NA	FAIL
Task 6		NA	FAIL
Task 6		NA	FAIL
Task 6		NA	FAIL
Task 6		NA	FAIL
Task 6		NA	FAIL
Task 6		NA	FAIL
Task 6		NA	FAIL
Task 7		NA	FAIL
Task 7		NA	PASS
Task 8		NOT GIVEN	NOT GIVEN
Task 9		NOT GIVEN	NOT GIVEN
Task 10		NOT GIVEN	UNCLEAR
Task 11		NOT GIVEN	NOT GIVEN
Task 12		NOT GIVEN	NOT GIVEN
Task 13		NOT GIVEN	NOT GIVEN
Task 14		NOT GIVEN	NOT GIVEN
Task 15		NOT GIVEN	NOT GIVEN
Task 16		NOT GIVEN	NOT GIVEN
Task S(1)		1/3	FAIL
Task S(1)		2/3	FAIL
Task S(1)		2/3	FAIL
Task S(1)		0/3	FAIL
Task S(1)		1/3	FAIL
Task S(1)		2/3	FAIL
Task S(1)		1/3	FAIL

Supplementary Figure 10. Connelly’s results by trial given per task, with green indicating a correct response, yellow indicating an unclear or unexpected response, and red indicating an incorrect response. In this case, the yellow trial in Task 5 is a mistrial by making a not “A-not-B” error, and in Task 10 by behaving unexpectedly. Also listed are control outcomes, with number correct out of the total given, and the outcome of each task.

HUXLEY	TRIALS	Controls	Outcome
Task 4		NA	PASS
Task 5		NA	PASS
Task 6		NA	PASS
Task 7		NA	PASS
Task 8		NA	PASS
Olf. Control		NA	PASS
Task 9		NOT GIVEN	NOT GIVEN
Task 10		NOT GIVEN	NOT GIVEN
Task 11		NOT GIVEN	NOT GIVEN
Task 12		NOT GIVEN	NOT GIVEN
Task 13		NOT GIVEN	NOT GIVEN
Task 14		NOT GIVEN	NOT GIVEN
Task 15		NOT GIVEN	NOT GIVEN
Task 16		NOT GIVEN	NOT GIVEN
Task S(1)		NOT GIVEN	NOT GIVEN
Task S(2)		NOT GIVEN	NOT GIVEN

Supplementary Figure 11. Huxley's results by trial given per task, with green indicating a correct response, yellow indicating an unclear or unexpected response, and red indicating an incorrect response. Also listed are control outcomes, with number correct out of the total given, and the outcome of each task.

PLATO	TRIALS	Controls	Outcome
Task 4		NA	PASS
Task 5		NA	PASS
Olf. Control		NA	PASS
Task 6		NA	FAIL
Task 6		NA	PASS
Task 7		NA	FAIL
Task 8		NOT GIVEN	NOT GIVEN
Task 9		NOT GIVEN	NOT GIVEN
Task 10		NA	UNCLEAR
Task 11		NOT GIVEN	NOT GIVEN
Task 12		NOT GIVEN	NOT GIVEN
Task 13		NOT GIVEN	NOT GIVEN
Task 14		NOT GIVEN	NOT GIVEN
Task 15		NOT GIVEN	NOT GIVEN
Task 16		NOT GIVEN	NOT GIVEN
Task S(1)		NOT GIVEN	NOT GIVEN
Task S(2)		NOT GIVEN	NOT GIVEN

Supplementary Figure 12. Plato's results by trial given per task, with green indicating a correct response, yellow indicating an unclear or unexpected response, and red indicating an incorrect response. In Task 5, the yellow box represents a mistrial by making one not "A-not-B" error. Also listed are control outcomes, with number correct out of the total given, and the outcome of each task.
